# Eudragit-Based Nanoparticles for Oral Drug Delivery

**DOI:** 10.3390/pharmaceutics18070813

**Published:** 2026-06-30

**Authors:** Filipa Bettencourt, Patrícia C. Pires, Francisco Veiga, Ana Cláudia Paiva-Santos, Amélia C. F. Vieira

**Affiliations:** 1 Department of Pharmaceutical Technology, Faculty of Pharmacy of the University of Coimbra, University of Coimbra, Pólo das Ciências da Saúde, Azinhaga de Santa Comba, 3000-548 Coimbra, Portugal; uc2020216888@student.uc.pt (F.B.); fveiga@ff.uc.pt (F.V.); 2 LAQV/REQUIMTE, Department of Pharmaceutical Technology, Faculty of Pharmacy of the University of Coimbra, University of Coimbra, Azinhaga de Santa Comba, 3000-548 Coimbra, Portugal; patricia.c.pires@ua.pt; 3 Department of Medical Sciences, University of Aveiro, 3810-193 Aveiro, Portugal; 4 Institute of Biomedicine—iBiMED, University of Aveiro, 3810-193 Aveiro, Portugal; 5 RISE-Health, Department of Medical Sciences, Faculty of Health Sciences, University of Beira Interior, Av. Infante D. Henrique, 6200-506 Covilhã, Portugal

**Keywords:** controlled drug release, Eudragit, nanoparticles, oral drug delivery, polymethacrylate

## Abstract

The development of oral drug delivery systems has become a major priority for pharmaceutical technology, driven by the growing demand for medicinal products that improve compliance, enhance therapeutic efficacy, and minimise drug-related adverse effects. Therefore, the ability to modulate drug release kinetics through systems capable of controlled and targeted delivery is crucial. In this context, Eudragit-based nanoparticles have demonstrated great potential in enhancing drug stability, controlling release profiles, and improving site-specific targeting in the gastrointestinal tract. Polymethacrylate copolymers (Eudragit^®^) exhibit pH-dependent solubility, mucoadhesive properties, and tunable drug-loading capacities, making them highly suitable for advanced oral formulations. This review provides a comprehensive analysis of the use of Eudragit^®^ in the design of nanoparticulate systems for oral drug delivery: inorganic nanoparticles, nanocrystals, lipid-based carriers, and polymeric nanoparticles. A special focus is given to the formulation’s composition, preparation method, physicochemical properties and the mechanisms of controlled drug release, but also to in vitro, ex vivo, and in vivo characterisation. Emphasis is placed on controlled-release strategies, targeted delivery, and the impact of polymeric materials in optimising therapeutic outcomes. By exploring these aspects, this review aims to highlight current research advances on Eudragit-based nanoparticles, their potential applications, and the challenges that must be addressed before these nanosystems can be considered robust platforms for improving oral drug bioavailability and efficacy.

## 1. Introduction

Oral delivery is the most preferred route of drug delivery, especially in the management of chronic disorders, due to its high patient compliance, cost-effectiveness, non-invasiveness, ease of use, and safety [1,2,3]. Ingestible dosage forms must be stable enough to withstand the harsh biochemical environment that characterises the gastrointestinal tract (GIT), effectively penetrate gastrointestinal tissues, enter the bloodstream, and deliver drugs to a biological target within an adequate timeframe and dosage window, ensuring the appropriate concentration for the expected clinical outcomes [1,4]. Notwithstanding, traditional dosage forms still present significant limitations, including low bioavailability due to compromised stability under varying pH conditions, enzymatic degradation, poor permeability, low site-specific accumulation, and limited mucoadhesion [4,5]. Thus, novel oral dosage forms aim to better meet patient requirements and therapeutic expectations by achieving site-specific targeting within the GIT while providing sustained and controlled release profiles [1].

Along with this change came drug carriers, which are defined as being a “system that can change the way the drug enters the body and its distribution in the body, control the release rate of the drug, and deliver the drug to the target organ” [6]. Among these, nanosized drug carriers are particularly advantageous as they enhance drug solubility due to the high surface area-to-volume ratio [2,7]. Nanotechnology has played a fundamental role in mitigating and overcoming some of the drawbacks associated with oral drug delivery and conventional treatments. It can be applied to the diagnosis, prevention, and treatment of various diseases, enabling some systems to function as theranostics [8]. Furthermore, nanoparticles (NPs) can be designed with biomaterials to design systems that protect the API from hydrolysis in the stomach, enabling it to be released in a sustained way at a targeted site [9].

On the other hand, there are polymers, such as the case of polymethacrylates, highly valuable in the design of oral dosage forms, as they are capable of enhancing drug stability and therapeutic efficacy. Although there are several commercially available polymethacrylates, Eudragit^®^ polymers are widely utilised and present in a wide range of commercial formulations due to their pH-dependent solubility, controlled-release properties, and ability to prolong drug loading [10].

The integration of Eudragit^®^ polymers with nanotechnology in the design of new formulations has been extensively investigated and may represent a key strategy for optimising final product quality.

This review aims to provide a comprehensive analysis of Eudragit-based NPs for oral drug delivery, focusing on different formulations and evaluating their specific potential applications and limitations. To achieve this, the review first describes the importance of enhancing oral drug delivery, with particular emphasis on controlled-release and targeted drug delivery mechanisms. Moreover, a detailed description of the distinct types of polymethacrylate copolymers and their specific properties is provided to elucidate their suitability for optimising oral drug release profiles. Further, by merging nanotechnology and Eudragit-based systems, an analysis of multiple drug nanodelivery systems with a focus on the APIs used, target sites, and therapeutic approaches is presented and discussed.

## 2. Controlled Drug Release and Targeted Drug Delivery

When discussing oral drug delivery, controlled release dosage forms are a “solid, semi-solid, solution or suspension designed to release active and/or inert ingredient(s) at a controlled rate” [11]. These dosage forms belong to the broader category of modified-release dosage forms, as they exhibit an intentionally altered release profile of their active and/or inert ingredients [12].

The concept of “drug delivery systems (DDS)”, which, although often used interchangeably with “dosage form”, represents a distinct idea. A DDS implies that there is a technology controlling the release rate of the drug [9,13]. Over the years, DDS have evolved significantly, allowing for precise targeting of drug concentrations within specific tissues and organs. This results in optimal drug concentration, accumulation in the desired target sites, extending the duration of the therapeutic effect, and minimisation of off-target distribution [9,13].

In the absence of periods where drug levels in the bloodstream are either sub- or supratherapeutic, patient convenience is enhanced, and adverse effects are minimised, resulting in increased safety [13]. These systems are used to improve drug pharmacokinetics, including controlling drug release kinetics, improving solubility, enhancing drug stability, overcoming biological barriers, and targeting drug delivery [9].

### 2.1. Kinetics of Controlled Drug Release

Release profiles can generally be split into three phases: the initial burst or lag phase, followed by the controlled release phase, ending with drug depletion and release rate tail-off [14]. Controlling the drug release rate in a system may be achieved through several mechanisms [9]. Concentration gradients play a significant role in determining the release rate across most controlled-release delivery systems. Diffusion, dissolution, swelling, affinity, and ion exchange are the primary mechanisms behind drug release control. Moreover, most controlled-release dosage forms are influenced by multiple mechanisms simultaneously [13].

In matrix-type systems, the drug molecules are uniformly dispersed or dissolved within the polymeric material forming the matrix, whereas in reservoir-type systems, the drug is surrounded by a permeable, porous, or non-porous membrane layer [9,13].

Diffusion-controlled systems tend to be either matrix-based (monolithic) or reservoir-based (water-insoluble polymeric membrane), where drug release is governed by Fick’s law (Case I) [15]. In a diffusion-controlled matrix, which refers to a non-swelling, noneroding drug-containing matrix, the release mechanism is primarily a concentration-driven release, where the rate-limiting step is drug diffusion [14]. In these matrix systems, it is common to have an initial burst release of the drug due to the rapid diffusion of surface-localised drug particles, followed by a first-order kinetics (Equation (1)), involving a progressively decreasing release rate throughout the process because the concentration gradient that drives diffusion decreases as the drug is depleted from the matrix. Characteristics like pore size, bond types, hydrophobicity, and thickness of the matrix will directly influence the dissolution rate, thereby affecting drug release [9,15].

(1) $$F=100(1-e^{\left(-K_{1}t\right)})$$

First order kinetic model (*F* = fraction of drug release; *k*_1_ = first order constant; *t* = time).

On the other hand, reservoir systems can present zero-order release kinetics (Equation (2)), characterised by a constant drug release rate following an initial equilibration phase. However, if membrane failure occurs, then dose dumping is observed [9,13,16,17].

(2) $$F=K_{0}t$$

Zero order kinetic model (*F* = fraction of drug release; *k*_0_ = zero order constant; *t* = time).

When diffusion is the main drug release mechanism, the Higuchi equation (Equation (3)) can be used to fit the release data (cumulative % drug release versus square root of time). This equation is derived from 2nd Fick’s law and is used when the release rate is determined by the concentration gradient [18,19].

(3) $$F=K_{H}t^{1/2}$$

Higuchi Kinetic model: (*F* = fraction of drug release; *K_H_* = Higuchi constant, *t* = time).

When the drug is dispersed in the matrix, the release process involves a dissolution step before diffusion can occur, which alters the release time dependency. In many controlled release systems, both dissolution and diffusion contribute to drug release [13,14]. In this case, the Korsmeyer–Peppas model (Equation (4)) is applied (log of % drug release versus log time), as it can be used as a non-Fickian model.

(4) $$F=K_{Kp}t^{n}$$

Korsmeyer–Peppas Kinetic model: (*F* = fraction of drug release; *K_kp_* = Korsmeyer–Peppas constant, *t* = time).

This model is widely used for polymeric systems analysis. In the equation, the *n* value (considering spherical geometry) can help to indicate the mechanism of release [18,20].

o *n* < 0.43—quasi-Fickian diffusion
o *n* = 0.43—Fickian diffusion mechanism
o 0.43 < *n* < 0.85—non-Fickian diffusion
o *n* = 0.85—case II transport (zero-order release)
o *n* > 0.85—super case II transport

(In case of thin films/slabs *n* values to be considered are *n* = 0.5 and 1, and in case of cylindrical samples *n* values to be considered are *n* = 0.45 and *n* = 0.89).

When referring to swelling as a controlled release mechanism, the materials forming the matrix/reservoir need to have some water-absorbing capacity. This is the case of matrix systems based on hydrophilic polymers, which have a drug release dependent on the rate at which water can effectively penetrate the matrix. If the water penetration rate is quicker when compared to polymer erosion, an increased degree of swelling will increase the drug release rate. On the other hand, water penetration into the matrix can depend on the time it takes for the polymeric network to relax. In this case, the drug release rate depends on the hydration rate of the polymer, making polymer relaxation and swelling the main forces for drug release. Such swellable systems are often associated with case II transport for drug release [14,17].

Finally, there can be cases where both swelling/relaxation and diffusion are present, resulting in anomalous transport [14,21]. In this case, in Korsmeyer–Peppas model, for spherical systems, anomalous transport is indicated by *n* value between 0.43 and 0.85 [20], as described before. Affinity-based systems leverage specific interactions between drug molecules and carrier materials to modulate the release kinetics. Such interactions are noncovalent, including electrostatic, van der Waals, hydrophobic, and hydrogen-bond interactions. For this reason, the drug’s release rate is dependent on the association constant of drug–ligand interactions. These release rates are, therefore, tunable [9,14]. Ion exchange systems rely on an ionic environment to obtain controlled release. The charged drug molecules are loaded into a resin, which is typically inert and can pass safely through the GIT. This system is especially suitable for enteral delivery [9].

### 2.2. Targeted Drug Delivery

Targeted delivery consists of selectively transporting a particular drug to a specific site, enabling therapeutic action to be secluded from other body areas and minimising potential adverse effects [22]. Targeted delivery can be attained passively or actively, by passive transport principles, the development of stimuli-responsive DDS, and via surface modification with specific targeting molecules. These stimuli-responsive systems are specifically engineered to selectively respond to internal or external stimuli, such as pH, temperature, biologically active molecules, heat, light, and ultrasound. Intrinsic stimuli are typically associated with pathological processes and can vary depending on the condition. The distinctive changes observed in pathological regions set them apart from normal healthy tissues. Consequently, a better understanding of local microenvironmental alterations in these areas has facilitated the development of advanced DDS capable of reacting to abnormal conditions, thus targeting drug delivery [23].

Passive targeting relies on the inherent properties of the DDS (shape, size, charge, and surface modifications), the physiological characteristics, and pathological changes in the target tissue. A careful balance of these parameters is required to design a DDS capable of interacting with biological barriers and tissues, directing the drug to diseased areas without the need for specific ligands or active recognition mechanisms. Particle size (PS) plays a central role in circulation kinetics and tissue penetration. Systems below 10 nm are rapidly eliminated by renal filtration, while those exceeding 200 nm are more prone to uptake by the mononuclear phagocyte system (MPS). This uptake can be potentially beneficial in different contexts: if the therapeutic strategy is to target macrophages, such as in inflammatory or infectious conditions, the enhanced MPS uptake is advantageous. For oral nanocarriers, an intermediate range (50–200 nm) is considered optimal, as it balances uptake across the intestinal mucosa, avoidance of rapid clearance, and sufficient stability to exploit passive targeting mechanisms after absorption. Morphology also influences passive targeting efficiency. While spherical NPs, such as vesicles and micelles, are most common, non-spherical morphologies, including rods, discs, stars, and filaments, have also been extensively explored. Depending on the morphology, NPs have been shown to display distinct hydrodynamic and cellular interaction profiles. For instance, when comparing polymeric micelles, filamentous ones are shown to remain in blood circulation longer than spherical ones. In dendritic cells, disc-shaped NPs have been shown to have higher membrane interaction, leading to enhanced phagocytosis compared to edged NPs. In epithelial cells, NP shape influences the internalisation mechanism. Optimisation studies of NP size and morphology can be conducted to evaluate the impact of independent formulation variables on these properties. Surface charge determines how the DDS interacts with physiological components. Cationic systems display enhanced interaction with negatively charged cellular membranes due to electrostatic interactions, promoting uptake. However, cationic NPs also exhibit higher cytotoxicity and faster clearance by phagocytosis (MPS). Negatively charged systems, while more compatible, are susceptible to serum protein adsorption, which can lead to their phagocytosis. Neutral NPs exhibit the longest circulation times due to reduced protein adsorption [24,25,26,27].

Solid tumours provide an example of how pathological physiology can be exploited in the context of passive targeting. Their vasculature is often disorganised, presenting irregular branching, heterogeneous perfusion, and extensive fenestrations. In parallel, inefficient lymphatic drainage hinders interstitial fluid clearance. Together, these alterations create a microenvironment that favours the passive accumulation of nanosized DDS. This phenomenon, commonly known as enhanced permeability and retention (EPR) effect, represents a passive targeting mechanism solely derived from the pathophysiological changes in the tumour environment [9]. EPR effect may contribute to the passive accumulation of NPS in colorectal tumours. However, oral colon targeting delivery also relies on gastrointestinal and tumour microenvironment-specific features such as pH and enzymes [28]. However, extravasation increases tumour interstitial fluid pressure, which disfavours the EPR effect [29,30].

In cases of inflammatory bowel disease (IBD), the increased permeability of inflamed intestinal tissues has been exploited as a passive targeting mechanism for NP-based drug delivery [31].

A different passive targeting approach is to design DDS with mucoadhesive properties, allowing them to interact and adhere to the mucus layer. Muchoadhesion takes place through electrostatic interaction between positively charged formulation and negative charge of the mucin, as well as through hydrogen bonding and hydrophobic interactions. It can also be enhanced by covalent interactions, such as disulfide bond formation using thiolated polymers [32,33]. On the other hand, when the aim is mucus penetration, the interaction with mucin should be reduced. This can be achieved through neutral or negatively charged polymers, which minimise the interactions with the mucin [33].

This can prolong residence time at the target site and enhance local drug exposure, which is advantageous for localised therapies such as inflammatory bowel disease. However, excessive mucoadhesion may immobilise formulation within the mucous layer, limiting diffusion toward the epithelium and reducing access to epithelial receptors or absorption sites [34]. Therefore, DDS design must balance mucus retention with mucus penetration, depending on whether the therapeutic goal is local mucosal treatment or systemic absorption across the epithelial barrier.

In active targeting, the DDS can interact with the desired target(s) due to the use of an affinity-based recognition sequence. Targeting can be receptor-mediated, transport-mediated or cell-specific (e.g., M cells), focusing on a unique tissue or molecule, which makes site-specific interactions extremely relevant [35,36].

The levels of receptors and transporters across the GIT also vary with the health state, and for this reason, can be used as targeting sites when overexpressed. For instance, the neonatal Fc receptor for IgG (FcRn) is overexpressed on intestinal epithelial cells during inflammatory disorders [37]. On the other hand, the apical sodium-dependent bile acid transporter (ASBT) present in epithelial cells decreases in Crohn’s disease (due to ileal inflammation and damage) but may increase in diabetes and obesity [38]. Also, peptide transporter 1 (PEPT1) increases in inflammatory bowel disease, diabetes and colorectal cancer [39,40]. Thus, for targeting, NPs require a ligand on their structure that is able to be recognised and specifically binds to an overexpressed receptor or transporter. Targeting ligands can be antibodies, proteins, peptides, aptamers, carbohydrates, or small molecules (e.g., bile acids) [9,14].

## 3. Polymethacrylates and Oral Drug Delivery

When choosing a material to be applied to a DDS for an intended administration route and site of action, biocompatibility, hydrophilicity, physical and protein binding properties, stability, degradability, pH, and temperature behaviours must be evaluated. Biomaterials are “materials destined to interface with biological systems to evaluate, treat, augment, or replace any tissue, organ, or function in the body” [9,41].

Polymers, with tunable chemistry, controllable and responsive properties, flexibility in conjugation and drug incorporation, and a wide variety of bulk compositions and physical properties, are the most widely used materials for DDS. They can be categorised as synthetic or natural; however, synthetics are more commonly used due to their predictable structure–function relationships, resulting in minimised batch-to-batch variations [9,42].

Polymer chemical structural analysis is useful for predicting possible degradation mechanisms, which primarily occur through hydrolysis, oxidation, photolysis, and proteolysis. However, the biological environment can also modulate polymer behaviour, and for this reason, degradation can occur intentionally or unintentionally. Surface properties are also critical and lead to different levels of protein adsorption, influencing organic reactions and controlled drug release [43].

Considering a DDS, polymer composition, molecular weight, and surface characteristics strongly influenced drug loading, encapsulation efficiency (EE), stability, and release behaviour [43,44,45,46]. When developing stimuli-responsive DDS, stimuli-responsive biomaterials are applied, and their behaviour is affected by the changes in the environment. These so-called “smart polymers” can respond to external or internal, physical or chemical stimuli [43]. Among stimuli-responsive biomaterials, pH-sensitive polymers are particularly relevant for oral delivery due to the marked pH variations encountered throughout the GIT. pH-sensitive polymers contain ionisable acidic or basic groups, capable of undergoing protonation or deprotonation in response to environmental pH changes, thereby altering polymer charge density, solubility, swelling behaviour, and permeability [23,43].

Polymers containing weak acidic groups are polyacids, and at acidic gastric conditions, their carboxylic groups remain predominantly protonated and less soluble, restricting premature drug release. At neutral and alkaline intestinal pH, ionisation promotes polymer swelling and drug release [47]. Conversely, polymers containing weak basic groups in their structure become protonated at low pH values, enabling distinct pH-dependent release behaviours [47,48]. Consequently, pH-responsive polymers enable site-specific drug protection and release within different regions of the GIT when pH-sensitive polymers are assembled into DDS membranes, their behaviour becomes even more versatile. These pH-sensitive membranes respond to environmental pH by undergoing physical or chemical changes that directly affect their permeability and drug release performance [49]. Two main mechanisms drive this response: pH-dependent ion transport and molecular recognition [47,50,51]. Changes in the protonation state of ionisable groups alter the charge density, leading to altered ion selectivity and permeability. In parallel, pH-dependent variations in non-covalent interactions between polymer chains and interacting molecules, such as drugs or biomolecules, regulate binding affinity and release kinetics. Collectively, these mechanisms enable environment-specific control of drug release across the GIT, although their in vivo performance may still be influenced by physiological variability in pH, transit time, and disease states [47,48].

Within this context, polymethacrylates have emerged as one of the most widely used classes of pH-responsive polymers in oral drug delivery. Their chemical versatility, tunable ionisation behaviour, and availability in multiple functional grades allow precise modulation of drug release profiles, making them particularly suitable for the design of gastro-resistant, intestinal, and colon-targeted delivery systems [52]. They are monographed in several Pharmacopoeia, including the European Pharmacopoeia and the USP/NF. According to the USP32-NF27, methacrylic acid copolymer is described as a fully polymerised copolymer of methacrylic acid and an acrylic or methacrylic ester [53]. They are commonly referred to as Eudragit, although various companies produce them under different names. For instance, Evonik Industries markets them as Eudragit, Colorcon calls them Acryl-EZE, Eastman Chemical refers to them as Eastacryl 30D, and BASF Fine Chemicals sells them under the name Kollicoat MAE [54]. Eudragit polymers are available in multiple grades, each distinguished by specific compositions, chemical properties, and pH-dependent solubility profiles, as outlined in Table 1. The differing ratios of their functional groups, such as methacrylates, methacrylic acid, and/or methacrylic acid esters in each polymer’s composition, determine their classification into cationic, anionic, or neutral [10]. This functional classification provides a more mechanistic understanding of their role in oral drug delivery than commercial nomenclature alone, as differences between grades primarily reflect variations in ionisable group content and polymer permeability, rather than fundamentally distinct release principles Moreover, the functional groups play a crucial role in modulating how and where drug release will occur [10,48].

Among the cationic grades, Eudragit E is commercially available in three different grades: Eudragit E100, Eudragit E 12.5 and Eudragit E PO. These grades are characterised by favourable properties such as strong adhesion, low viscosity, high pigment-binding capacity, and minimal polymer weight gain. These polymers are typically employed to enable immediate gastric drug release and taste-masking [52,54,55]. Eudragit E is an aminoalkyl methacrylate copolymer that is highly soluble below pH 5. In acidic media, protonation of its tertiary amine groups promotes polymer dissolution. Thus, drug release from Eudragit-based systems is expected to be mainly dissolution-controlled [56]. The taste-masking property is derived from the polymer’s insolubility in the neutral oral cavity pH, preventing drug release in the mouth [57]. Thus, these polymeric grades can increase the solubility and bioavailability of enterically insoluble compounds with unpleasant taste [58].

Regarding anionic Eudragit polymers, they include Eudragit L and S and FS. Eudragit L, S and FS are anionic methacrylic acid copolymers that ionise and dissolve at intestinal pH. Drug release from formulations based on these polymers is mainly driven by pH-dependent dissolution [54].

The Eudragit L series includes four commercial grades: L100, L100-55, L12.5, and L30D-55, while the Eudragit S series comprises S12.5 and S100 [10]. Eudragit L100 (EL100) remains insoluble in gastric pH, enabling indirect targeted drug delivery to the upper small intestine. Due to its commercially available physical form, it is commonly used for film coating and as a matrix former in controlled release and gastro-resistant dosage forms [59,60,61,62]. On the other hand, Eudragit S100 (ES100) is designed for enteric coating applications aimed at the colon (pH 7), remaining insoluble in gastric and upper intestinal fluids [59,60,63]. When this polymer is used to coat cationic NPs, its dissolution in the GIT enables re-exposure of the underlying positively charged NP surface. This enables electrostatic adhesion to negatively charge cell membranes and improves NP retention [64]. Eudragit FS (grades FS30D and FS100) presents a pH-dependent solubility similar to ES100; however, their glass transition temperatures make them more suitable carriers for hot melt extrusion processes [65].

In the Handbook of Pharmaceutical Excipients, Eudragit RL and RS are classified as neutral polymers, and the USP32 classifies them as ammonium methacrylate copolymers. Both consist of acrylic acid and methacrylic acid esters, but according to USP32-NF27, the key distinction between them lies in the ratio of ammonium methacrylate units [53,54]. The quaternary ammonium groups present in their backbone remain permanently ionised, therefore conferring a weak cationic charge to the matrix. Since Eudragit RL has a higher proportion of these functional groups than RS, it exhibits greater aqueous permeability. Nonetheless, Eudragit RL100 and RS100 are pH-independent ammonium methacrylate copolymers and remain insoluble across the physiological pH range of the GIT. Their quaternary ammonium groups allow gradual fluid penetration and polymer swelling, subsequently enabling sustained drug release mainly by diffusion through the permeable polymeric network. Optimisation of controlled release profiles can be attained using these two polymers in different ratios [66].

Eudragit NE (30 D and 40) and NM (30D) represent neutral, swellable, pH-independent polymers. Drug release from NE/NM systems is mainly controlled by water penetration and diffusion through the polymeric network [10,54].

Thiolated derivatives of Eudragit have been increasingly employed to overcome the intrinsic limitations of the unmodified polymer in oral nanoparticulate delivery, namely premature drug leakage and insufficient mucoadhesion. The introduction of free thiol groups into the polymer backbone can promote disulphide crosslinking with other thiolated polymers, enhancing their stability in gastric conditions and enabling selective redox-responsive cleavage in physiologically reducing environments, such as the colon, tumour tissues, or intracellular compartments. This chemical modification has proven particularly effective in combination systems, where thiolated Eudragit interacts with thiolated polymers such as sodium alginate, dextran, pectin, or chitosan to produce thiolated crosslinked NPs that can be activated and promote drug release in reducing media, as discussed in Section ‘Thiolated Nanoparticles’ [67,68,69,70,71].

Studies have suggested that certain Eudragit grades may alter apparent modulate P-glycoprotein (P-gp)-mediated transport activity. For instance, a study conducted on Caco-2 cells studied the effect of ES100, EL100 and E RL 100 on the permeability of a P-gp substrate (rhodamine-123) and concluded that ES100 had a significant impact on the accumulation of P-gp substrate and also reduced the expression of P-gp [72]. In another study, described in Section 4.1.1, mesoporous silica NPs (MSNPs) were coated with ES100, and permeability studies conducted in Caco-2 cell monolayer models demonstrated an improvement of meropenem permeation when this was encapsulated, wherein it increased 1.9-fold and 2.4-fold in NPs without and with coating, respectively. Also, the study reports a decrease in the secretory transport and a reduction in drug efflux ratios in NPs coated with ES100, when compared with their uncoated counterparts, wherein this decrease was higher in coated NPs [73].

The reduced efflux ratios observed in Caco-2 cell monolayer models with Eudragit-coated formulations may reflect not only transporter modulation, but also alteration of drug release kinetics, increased epithelial contact time, or other formulation-dependent permeability effects. Accordingly, the current data support only a possible in vitro interaction with P-gp-associated transport, while its relevance to intestinal absorption in vivo remains unproven.

In vivo, a study using tacrolimus-loaded nanocapsules coated with a mixture of Eudragit RS and Eudragit L100-50 (EL100-55) demonstrated enhanced drug’s oral bioavailability by 4.9-fold in rats and 2.45-fold in pigs. This was possibly due to the ability of NPs to escape the P-gp-mediated intestinal efflux, although the effect was not attributed directly to the coating composition alone [74].

Thus, although Eudragit-based systems may change P-gp activity, the absence of specific studies prevents any conclusion that these polymers meaningfully inhibit P-gp in a translational sense. Therefore, it remains uncertain whether similar behaviour would be observed in clinical studies [72,73,74]. Further in vivo studies are required to confirm whether these polymers can enhance the intracellular accumulation and intestinal permeability of P-gp substrates, and whether this effect can be exploited to improve drug delivery or targeting.

**Table 1:** Description of several commercially available Eudragit grades.

Grade		Chemical Name	pH-Dependent Solubility	Glass Transition Temperature (°C)	Physical Appearance	Applications	Oral Safety Information (FDA)	Reference
**Cationic**	Eudragit E100	Poly (butyl methacrylate, (2-dimethylaminoethyl) methacrylate, methyl methacrylate) 1:2:1	Soluble in gastric fluid below pH 5	48	Granules	Increased bioavailability and dissolution profile; high oral bioavailability.	MPD = 4.57 mg *	[10,52,54,55,75,76,77,78,79,80]
	Eudragit E PO				Powder			
	Eudragit E 12.5			-	Organic solution (12%)			
**Anionic**	Eudragit L 100	Poly (methacrylic acid, methyl methacrylate) 1:1	Soluble in intestinal fluid from pH 6	150	Powder	Increased oral absorption, increased taste masking, controlled release, enteric targeted drug delivery, delayed release profile, high oral bioavailability.	MPD = 10.08 mg **	
	Eudragit L 12.5			-	Organic solution			
	Eudragit L 100-55	Poly (methacrylic acid, ethyl acrylate) 1:1	Soluble in intestinal fluid from pH 5.5 (resistant to gastric juice but readily dissolves at pH above 5.5)	110	Redispersible powder	Enteric coating	MPD = 15 mg **	
	Eudragit L30 D-55			-	Aqueous dispersion (30%)			
	Eudragit S100	Poly (methacrylic acid, methyl methacrylate) 1:2	Soluble in intestinal fluid from pH 7	150	Powder	Increased oral absorption, increased taste masking, controlled release, colonic-specific drug delivery, delayed release profile, high oral bioavailability.	MPD = 4.32 mg **	
	Eudragit S12.5				Organic solution (12.5%)			
	Eudragit FS100	Poly (methyl acrylate, methyl methacrylate, methacrylic acid) 7:3:1	Soluble in intestinal fluid from pH 7	55.6	Granules	Increased oral absorption, increased taste masking, controlled release, colonic-specific drug delivery, delayed release profile, high oral bioavailability.		
	Eudragit FS30 D			48	Aqueous dispersion (30%)			
**Neutral**	Eudragit RL100	Poly (ethyl acrylate, methyl methacrylate, trimethylammonioethyl methacrylate chloride) 1:2:0.2	Insoluble High permeability, pH-independent swelling	70	Granules	Sustained release, improved permeation and increased bioavailability and shelf life.		
	Eudragit RLPO				Powder			
	Eudragit RL30D			-	Aqueous dispersion (30%)			
	Eudragit RL12.5			-	Organic solution (12.5%)			
	Eudragit RS100	Poly (ethyl acrylate, methyl methacrylate, trimethylammonioethyl methacrylate chloride) 1:2:0.1	Insoluble Low permeability pH-independent swelling	64	Granules		MDE = 37–38 mg *	
	Eudragit RS PO				Powder			
	Eudragit RS 30D				Aqueous dispersion			
	Eudragit RS 12.5				Organic solution			
	Eudragit NM 30D	Poly (ethyl acrylate, methyl methacrylate) 2:1	Insoluble Low permeability pH-independent swelling		Aqueous dispersion (30%)			
	Eudragit NE 30D				Aqueous dispersion (30%)			

MDE: Maximum Daily Exposure; MPD: Maximum Potency per Unit Dose. * https://www.fda.gov/media/182874/download (accessed on 24 May 2026) (2024); ** https://www.fda.gov/media/186268/download (accessed on 24 May 2026) (2025).

## 4. Nanoparticles Containing Eudragit for Oral Drug Delivery

According to the Data Bridge Market Research report, the “oral delivery market is expected to witness market growth at a rate of 7.60% in the forecast period of 2022 to 2029” [81]. Nanotechnology has emerged as a transformative field in the realm of medicine and drug design, particularly in oral drug delivery, playing a vital role in addressing the associated challenges. It has facilitated the incorporation of increasingly complex APIs at a reduced dimensional scale [82]. PS is a critical factor in various stages of drug formulation development and delivery. It will directly influence stability, formulation feasibility, and the ability to overcome biological barriers for absorption. Reducing PS increases surface area, thereby enhancing interfacial solubility, adhesion, and interaction with cell membranes [83]. Consequently, nanoscale size reduction is very advantageous for oral delivery systems. Beyond this, nanotechnology further enhances therapeutic efficacy by increasing bioavailability, enabling extended and controlled drug release, protecting active compounds from degradation, and facilitating site-specific targeting [82].

To ensure optimal performance, effectiveness, and broad applicability, nanocarriers must possess specific properties. Such properties include high traceability and imaging capability, dispersibility, specificity and selective cellular binding, substantial cargo-loading capacity, biocompatibility, and minimal toxicity [84]. The following section provides a detailed explanation of Eudragit-based NPs, and Table 2 summarises studies on their application. Indeed, in the context of NPs, Eudragit can be used either as the main polymer matrix in polymeric NPs or as a coating applied to preformed NPs, including inorganic NPs, nanocrystals, lipid NPs, and performed polymeric NPs.

### 4.1. Inorganic Nanoparticles

Inorganic NPs (INPs) constitute a varied class of nanomaterials, including metal, metal oxide, and silica-based systems. Their nanoscale dimensions confer unique electronic, magnetic, catalytic, and optical properties enabling applications to be antibacterial and anticancer agents, biosensors, and regenerative or imaging tools. However, they present potential toxicity, urging the need for precise inorganic nanoparticulate systems to optimise safety and efficacy [98].

#### 4.1.1. Mesoporous Silica Nanoparticles

MSNPs a subclass of INPs, are distinguished by their biocompatible, low toxicity, high internal surface area, tunable pore structure, and high loading capacity (LC) of both hydrophilic and hydrophobic drugs [85,99,100]. These characteristics enable the encapsulation of a wide range of therapeutic agents, including small molecules, peptides, and genetic material, while also shielding them from degradation and immune system recognition [99]. Furthermore, the presence of surface silanol groups enables chemical functionalization, making it possible to have sustained drug release profiles, pH stimuli-responsive behaviour, and cell targeting, and higher drug loading [100]. Amino modification of MSNPs is very commonly observed, as it introduces a basic character to the particle, allowing for the formation of amide bonds. with other functional groups, such as, for example, carboxylic acids [101,102,103]. Kassem et al. [85] developed catechin-loaded MSNPs, with the objective of enhancing the solubility of this flavonoid and improving its oral bioavailability [104]. The authors prepared colon-targeted, catechin-loaded amino-functionalised MSNPs coated with ES100 (NH2-MSNPs/CHT@EUS-100) to enable pH-controlled, site-specific release. The ES100 coating was additionally intended to prolong the residence time of the drug at the colonic site. It was observed that amino grafting of MSNPs led to an increase in Zeta potential (ZP), rendering the particles more positively charged. This favoured electrostatic attraction with the negatively charged ES100, thereby facilitating the coating process and enhancing its stability. In addition, amino groups also functioned as additional binding sites for catechin (CHT), both through hydrogen bonding with its acceptor groups and electrostatic interactions with the phenolic hydroxyl moieties, resulting in higher drug loading and adsorption efficiency compared with non-functionalised MSNPs, despite the latter possessing larger pore volume and surface area.

The release behaviour was assessed, under a simulated gastrointestinal pH gradient, progressing from gastric (pH 1.9) to intestinal (pH 5.5) and colonic (pH 7.4) conditions, at 2 and 5 h intervals. Free CHT rapidly dissolved, with nearly 95% of the compound being released within the first 2 h. In contrast, CHT encapsulated in MSNPs (MSNPs/CHT) demonstrated a slower, more controlled release, achieving approximately 79% within the same timeframe. NH2-MSNPs/CHT@EUS-100 exhibited the most pronounced drug retention, with negligible release during the gastric stage. This stronger retention was attributed not only to the reduced pore dimensions and volume of aminated MSNPs, but also to the stronger intermolecular interactions established between CHT and the amino-functionalised silica surface. Nevertheless, an initial burst release was noted in both CHT-loaded MSNPs and NH2-MSNPs/CHT@EUS-100 at pH 1.2, likely associated with surface-localised or incomplete confined CHT molecules. This was followed by a slower, diffusion-driven release attributed to the presence of adsorbed CHT molecules in the internal silica network. Importantly, the ES100 coating imparted pronounced pH-responsiveness, with less than 2% of CHT being released under acidic conditions, increasing only slightly to 4% at pH 5.5. This modest release below the dissolution threshold of ES100 may be attributed to partial water uptake and restricted polymer swelling, which can increase free volume and facilitate minor diffusion of surface-located drug fractions without compromising coating integrity. This behaviour has been previously reported for ES100 coatings, where drug transport under non-dissolving conditions is governed by diffusion through hydrated polymer networks rather than polymer dissolution [105,106]. On the other hand, a sharp release of approximately 90% occurred under colonic pH after 5 h. This lag time indicated that ES100 successfully shielded the drug from premature release in the upper GIT, thereby ensuring colonic targeting. CTH release from CAT-loaded MSNPs and from catechin-loaded NH2-MNSPs (NH2-MSNPs/CHT) was consistent with Fickian diffusion, as indicated by fitting the Higuchi equation and the Korsmeyer–Peppas model. In contrast, the release of CTH from NH2MSNPs/CHT@EUS-100 follows a supercase II transport (*n* = 1.886 with Korsmeyer–Peppas model). This indicates that after coating, drug release became predominantly regulated by polymer swelling and relaxation following ionisation of the methacrylic acid moieties at neutral pH. Thus, the ES100 coating not only delayed release temporally, but fundamentally altered the release mechanism of the system [85].

**Figure 1:**
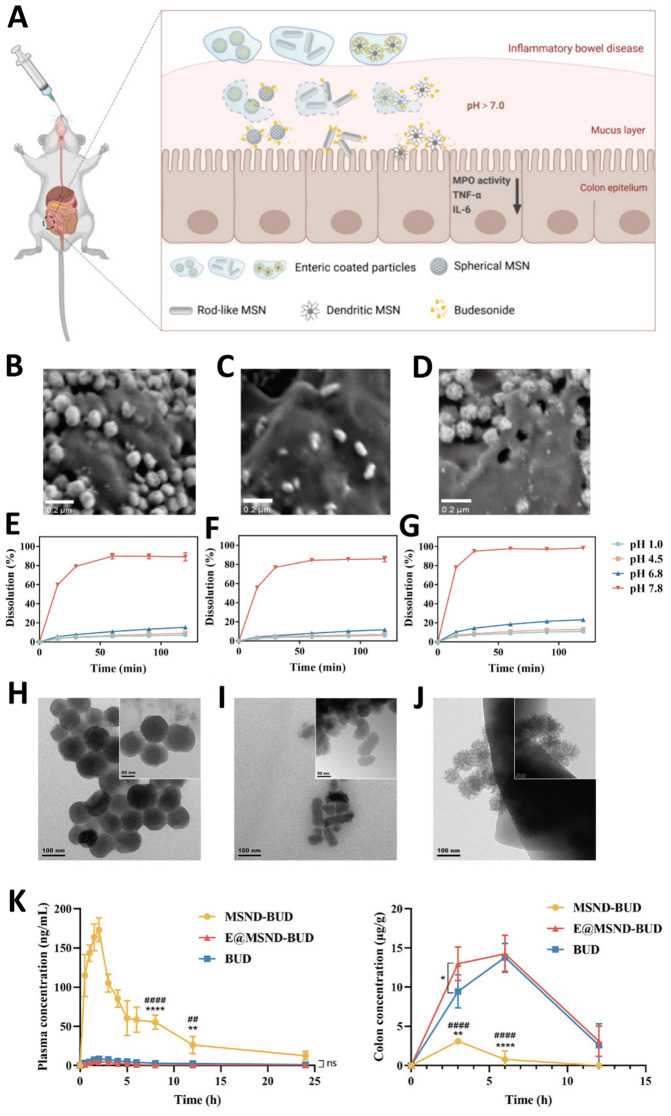
(**A**)—Schematic representation of the developed NP systems, including the intended behaviour under colonic conditions; (**B**)—SEM image of the developed spherical MSNPs; (**C**)—SEM image of the developed rod-shaped MSNPs; (**D**)—SEM image of the developed MSNDs; (**E**)—in vitro drug release profiles of the developed spherical MSNPs; (**F**)—in vitro drug release profiles of the developed rod-shaped MSNPs; (**G**)—in vitro drug release profiles of the developed MSND; (**H**)—TEM image of the residue of the developed spherical MSNPs after in vitro drug release assessment at pH 7.8; (**I**)—TEM image of the residue of the developed rod-shaped MSNPs after in vitro drug release assessment at pH 7.8; (**J**)—TEM image of the residue of the developed MSND safter in vitro drug release assessment at pH 7.8; (**K**)—in vivo pharmacokinetic study results (left—plasma; right—colon), Statistical significance between MSNDs-BUD and BUD is represented by **, **** for *p* < 0.01 and *p* < 0.0001, respectively. Statistical significance between E@MSNDs-BUD and BUD is represented by ##, #### for *p* < 0.01 and *p* < 0.0001, respectively; (BUD: budenoside; E@MSND-BUD: enteric-coated MSNDs-BUD; MSNDs: dendritic MSNPs; MSND-BUD: BUD-loaded MSNDs; MSNPs—mesoporous silica NPs; NPs—nanoparticle); adapted from permission from Li et al. [86]. Copyright 2026 American Chemical Society.

In the context of IBD, application of Eudragit polymers can play an important role, contributing to the localised action and higher drug concentrations in colonic tissue. Li et al. [86] synthesised NPs that were loaded with budesonide (BUD) and coated with ES100 (Figure 1A). Three different shapes of MSNPs were produced: spherical, rod-shaped, and dendritic (Figure 1B–D). ZP evaluation exhibited similar values for all particles; however, dendritic MSNPs (MSND) showed superior drug LC and EE, likely due to their larger pore entrances, greater pore accessibility, and higher surface area, which facilitated BUD diffusion into the mesoporous network. To study the release behaviour of BUD from the NPs, dissolution studies were conducted in simulated gastrointestinal pH conditions (pH 1.0, pH 4.5, pH 6.8, pH 7.8) (Figure 1E–J). At pH 1.0, drug release was minimal (less than 10%), while in alkaline conditions, rapid release was observed, exceeding 80% within 30 min. Among the three morphologies, MSNDs showed the highest cumulative release across the tested pH range, while rod-shaped particles consistently exhibited the lowest. Morphological analysis revealed that rod-like MSNPs had an elongated morphology with fewer pores, which contributed to a slower drug diffusion rate. In contrast, MSNDs featured an extensive porous network with a large surface area, facilitating faster dissolution and more efficient drug release. After the oral administration of the MSNPs suspensions in a murine model of IBD, the colonic tissue was analysed using in vivo imaging systems. It was concluded that after degradation of the Eudragit layer, MSNDs achieved the highest accumulation in the inflamed colon, followed by rod-shaped and spherical particles. This was attributed to the dendritic architecture, which has a branched, fibrous surface that enhances mucoadhesion, allowing for more efficient penetration through the dense mucous barrier, contributing to prolonged mucosal retention, and slowing mucosal clearance. Pharmacokinetic results (Figure 1K) indicated that low systemic absorption of BUD was observed after administration of both coated and uncoated MSNPs, supporting the hypothesis of a localised therapeutic effect. It was observed that MSNPs coated with ES100 elicited a significantly higher anti-inflammatory response compared to free BUD, while uncoated MSNPs did not show comparable therapeutic benefits. The highest effectiveness of MSNDs with Eudragit is likely due to their enhanced accumulation at the target site, sustained drug release profile, and improved colonic retention, all of which resulted in better therapeutic outcomes, with reduced adverse effects. MSNDs without coating failed to demonstrate therapeutic efficacy, likely because the absence of a protective polymer led to premature release of BUD from the mesopores, reducing the drug’s availability at the inflammation site [86].

Also, for the treatment of IBD, Qu et al. [87]. developed pH-responsive, amino-modified MSNPs highly loaded with prednisone (Pred) and BUD, respectively. Unmodified MSNPs initially displayed a negative ZP, which shifted to positive values after functionalisation with amino groups. Subsequent coating with ES100 effectively masked the surface charge, leading to an overall negative ZP, demonstrating that ES100 was successfully deposited onto the surface of amino-functionalized MSNPs. Using the same method employed by Kassem et al. [86], in vitro drug release results showed that both free Pred and amino-modified MSNPs containing Pred (Pred-A-MSNPs) demonstrated a rapid dissolution profile, with approximately 86% of the drug being released within the first 2 h under acidic conditions. In contrast, Eudragit-coated MSNPs (Pred-A-E-MSNPs) displayed a more controlled release, with only 18% of prednisolone being released at pH 1.9 during the initial 2 h. Then, at pH 7.4, the NPs showed a cumulative release of 78% was observed over 24 h, indicating a pH-sensitive release mechanism inherent to the formulation. For BUD, the non-encapsulated drug displayed a 56% release within the first 2 h, and reached 71% after 24 h. Its limited release rate is attributed to its poor aqueous solubility. The release profile of amino-modified MSNPs loaded with BUD (BUD-A-MSNPs) resembled that of the free drug, with an overall release of approximately 80% within 5 h. Conversely, Eudragit-coated MSNPs (BUD-A-E-MSNPs) demonstrated pH-dependent behaviour: only 12% of the drug was released at pH 1.9 during the first 2 h, followed by an additional 7% at pH 5.5. Upon elevation of the pH to 7.4, around 60% of BUD was released, reflecting a release pattern analogous to that observed with Pre-A-E-MSNPs. In vivo experiments were conducted, using a murine model of colitis induced by dextran sulphate sodium (DSS), to investigate the therapeutic potential of BUD-loaded NPs. This study was not conducted with prednisolone, since it, according to the authors, was not able to reverse inflammation using the same model. All treatment groups, including free Bud, BUD-A-MSNPs, and BUD-A-E-MSNPs, showed improvements in Disease Activity Index (DAI) scores compared to the untreated DSS group, with the nanoparticulate formulations having the most impactful results. In the proximal colon, no significant differences in therapeutic outcomes were noted between BUD-A-MSNPs and BUD-A-E-MSNPs compared to the untreated DSS colitis group. In the distal colon, however, both groups treated with MSNPs exhibited improved drug delivery efficiency, resulting in less tissue damage compared to the untreated DSS group and the free drug treatment. The authors noted that incomplete dissociation of Eudragit, possibly due to the lower pH from intestinal inflammation, may have limited drug release. To confirm targeted delivery and its biological effects at the molecular level, quantitative reverse transcription PCR analysis was conducted on colonic tissues. The results showed that treatments with BUD-A-MSNPs and BUD-A-E-MSNPs modulated gene expression related to inflammation and significantly boosted the anti-inflammatory effects of BUD, compared with the free drug. These findings indicate that NP-based delivery systems, whether coated or uncoated, enhance drug targeting and therapeutic effectiveness in colitis models. However, the presence of Eudragit may further contribute to stability, site-specific release, and pharmacokinetic advantages [87].

**Figure 2:**
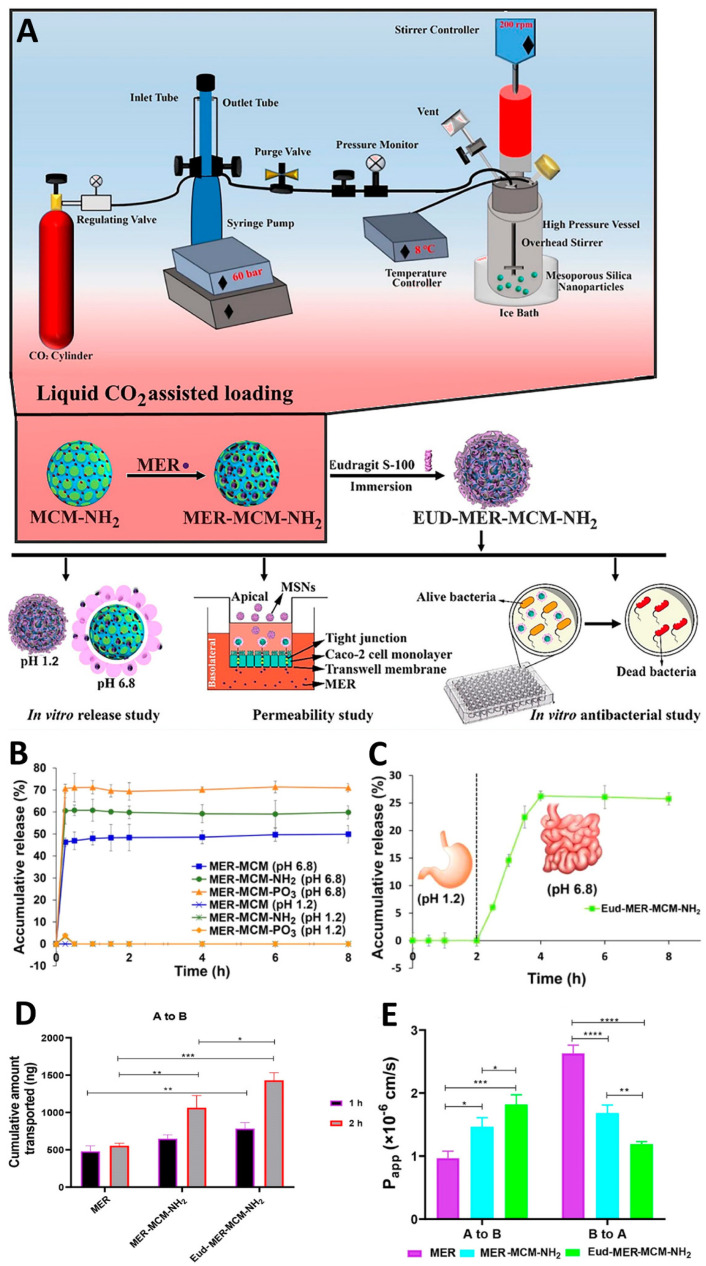
(**A**)—Schematic representation of the developed nanosystem’s composition, preparation method, and performed assays; (**B**)—in vitro drug release results from the different developed formulations (MER-MCM; MER-MCM-NH_2_; MER-MCM-PO_3_), at different pH levels; (**C**)—in vitro drug release results from Eud-MER-MCM-NH_2_ NPs in universal buffer, first at gastric pH for 2 h, and then at intestinal pH for 6 h; (**D**)—in vitro drug permeation results, showing the cumulative amount of MER transported from A to B; (**E**)—apparent permeability coefficient (P_app_) of the developed formulations; Statistical significance is represented by *, **, ***, **** for *p* < 0.01; *p* < 0.0036, *p* < 0.0002, *p* < 0.0001, respectively. (Eud-MER-MCM-NH2: Eudragit coated MER-MCM-NH_2_; MCM—mesoporous silica NPs MCM 41; MER: meropenem; MER-MCM: MER-loaded-MCM; MER-MCM-NH_2_: MER-loaded-amino-functionalised MCM; MER-MCM-PO_3_: MER-loaded-phosphonate-functionalised MCM); adapted with permission from Raza et al. [73]. Copyright 2026 American Chemical Society.

Raza et al. [73] developed pH-responsive MSPNPs loaded with meropenem (MER). MER, a broad-spectrum carbapenem antibiotic, is usually administered parenterally, either through intermittent bolus or continuous infusion, to prevent degradation in aqueous media and maximise therapeutic plasma concentrations. However, bolus infusion often leads to sub-therapeutic levels, falling below the minimum inhibitory concentration. Additionally, due to its short half-life and rapid renal clearance, MER requires frequent dosing, typically three times daily, which imposes a significant burden, both because of the need for repeated drug preparation and reconstitution, and also since more frequent dosing is often linked to lower patient compliance. Furthermore, owing to its hydrophilic nature, MER has limited oral bioavailability. Although prodrugs have been proposed to overcome this limitation, economic and manufacturing challenges make this approach commercially unfeasible. As MER is thermolabile, the authors used liquid CO_2_ as an alternative method for drug incorporation. ES100 served as a coating agent, shielding the drug from acidic gastric conditions and aiming at targeted release in the intestinal environment. The MSNPs were successfully synthesised and functionalised with either phosphonate (MER-MCM-PO_3_) or amine terminal groups (MER-MCM-NH_2_), followed by MER loading (Figure 2A). Although MER-MCM-PO_3_ exhibited a higher drug LC, this surface modification did not support subsequent coating, as it failed to confer MSNPs’ a positive surface charge. Therefore, despite their comparatively lower LC, MER-MCM-NH_2_ were selected for further coating with ES100 (Eud-MER-MCM-NH_2_). In vitro drug release studies (Figure 2B,C) demonstrated that Eud-MER-MCM-NH_2_ exhibited pH-responsive release behaviour, with no drug release at gastric pH, and sustained release under intestinal conditions, effectively protecting MER from gastric degradation, and enabling controlled drug release at simulated intestinal pH. In vitro permeability assays (Figure 2D,E) were performed using the Caco-2 monolayer culture model and evidenced that both MER-MCM-NH_2_ and Eud-MER-MCM-NH_2_ significantly enhanced MER’s absorptive transport. The improved permeation was higher for Eud-MER-MCM-NH_2_ (around 2.4-fold) than for MER-MCM-NH_2_ (1.9-fold) [73].

The study showed pH protection, enhanced permeability, and efficient uptake in multiple cell lines, but there were no in vivo pharmacokinetic or efficacy studies, lacking the confirmation that improved epithelial transport translates into improved oral bioavailability, and that NPs remain stable in the dynamic gastrointestinal environment. Additionally, the increment in absorptive transport and decrease in secretory is potentially important because it suggests altered interaction with epithelial barriers, possible modulation of efflux transporters, and prolonged intracellular retention. Furthermore, this enhanced permeability was not demonstrated mechanistically. Adding to this, a marked discrepancy between dry-state PS and hydrodynamic behaviour, together with the absence of in vivo validation, raises important questions regarding colloidal stability, physiological dispersion, and translational reproducibility.

#### 4.1.2. Nanodiamond-Based Nanoparticles

Nanodiamond-based NPs (NDNPs) present good biocompatibility and lower toxicity when compared with other carbon-based nanomaterials, such as carbon black, carbon nanotubes, or quantum dots. Their characteristic sharp edges facilitate endosomal membranes’ penetration, benefiting the cytosolic delivery of several drugs, including antitumour ones. Nonetheless, the strong covalent bonding within the diamond framework renders NDNPs chemically inert, which limits the possibility for subsequent modifications, requiring more specialised approaches [64,107].

**Figure 3:**
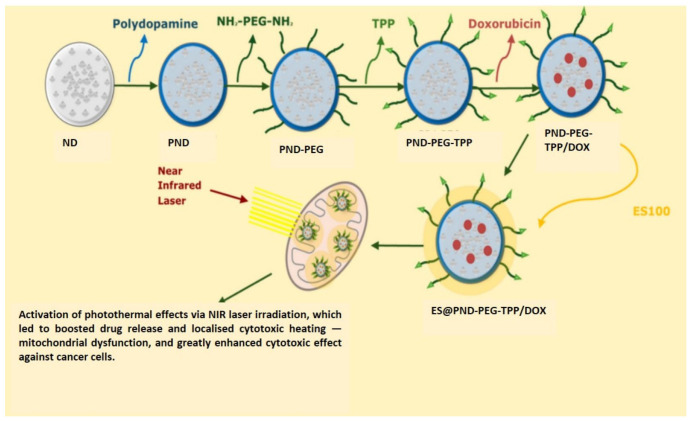
Schematic representation of doxorubicin-loaded NDNP coated with Eudragit S100, including the intended anticancer mechanism of action. Eudragit S100−coated nanodiamond-based NPs as an oral chemo-photothermal delivery system for local treatment of colon cancer. (DOX: Doxorrubicin hydrochloride; ES@PND-PEG-TPP/DOX: Eudragit-coated PND-PEG-TPP/DOX; ND: nanodiamond; PEG: polyethylene glycol; PND: ND coated with polydopamine; PND-PEG: PEG-functionalized PND; PND-PEG-TPP: TPP-functionalized PND-PEG; PND-PEG-TPP/DOX: Doxorrubicin-loaded- PND-PEG-TPP. Developed using PowerPoint, adapted from [64].

Su et al. [64] developed NDNPs designed for the targeted delivery of DOX to colorectal tumours (Figure 3). Due to the strong covalent bonds between carbon atoms of NDNPs, the authors incorporated polydopamine as a nanocarrier. Its structure, rich in aromatic rings, allows efficient adsorption of anthraquinone-based antitumour agents on its surface, and it also offers notable photothermal conversion abilities. This dual functionality creates a synergistic effect between chemotherapy and photothermal therapy. For better targeting and adhesion to the colon, the researchers used diamino polyethylene glycol as a linker (NH_2_−PEG−NH2) and triphenylphosphonium (TPP) as a cationic surface charger with high lipophilicity, which aids in mitochondrial targeting. To protect the nanocarrier from degradation in the stomach, prevent adhesion and absorption in the small intestine ES100 was used as a protective coating- It allows dissolution in the colonic environment, and hide nanocarrier positive charge. Once inside colon cancer cells, these NPs can be directed to the mitochondria because of the presence of TPP. The activation of photothermal effects via Near-Infrared (NIR) laser irradiation can then boost drug release and cause localised cytotoxic heating, which can lead to mitochondrial dysfunction, and greatly enhance the cytotoxic effects against cancer cells. Figure 3 schematically illustrates the rationale behind these NPs’ formulation and design assembly.

In vitro release assays of uncoated (PND-PEG-TPP/DOX) and coated (ES@PND-PEG-TPP/DOX) nanodiamonds were performed using simulated gastrointestinal fluids. The cumulative drug release of PND-PEG-TPP/DOX and ES@PND-PEG-TPP/DOX was 55.09% and 40.34%, respectively. Under laser irradiation, drug release increased to 75.31% for PND-PEG-TPP/DOX and 51.99% for ES@PND-PEG-TPP/DOX. These results suggest that the ES100 coating effectively reduced premature release in the upper GIT and that NIR laser irradiation can promote enhanced drug release. The colonic delivery of DOX was indicated by PS modification. In SGF and SIF, ES@PND-PEG-TPP/DOX showed no significant changes in PS (221 nm), whereas in SCF, a clear PS reduction (smaller than 190 nm after 2 h) was observed, indicating ES100 dissolution and DOX release. In vitro fluorescence imaging revealed minimal fluorescence signals for free DOX and PND-PEG-TPP/DOX, predominantly in the liver. On the other hand, ES@PND-PEG-TPP/DOX exhibited an even significantly lower fluorescence, compared to the former, which was suggestive of effective mitigation of DOX’s release prior to reaching the colon, reducing intestinal absorption, and thereby decreasing systemic side effects. Regarding retention in the GIT, ES@PND-PEG-TPP/DOX exhibited stronger fluorescence signals in the small intestine and colon, when compared with free DOX and PND-PEG-TPP/DOX. Furthermore, in vivo tumour therapy efficacy studies, performed on Balb/c mice bearing colon cancer tumours, highlighted that ES@PND-PEG-TPP/DOX combined with NIR laser radiation had the strongest growth inhibition rate on tumours [64].

### 4.2. Nanocrystals

Nanocrystals (NCs) are crystalline particles with dimensions below 100 nm, comprising a carrier-free nanotechnology, as the drug itself forms the matrix, thereby eliminating the need for large amounts of excipients. NCs improve the bioavailability of poorly water-soluble drugs, as they enhance dissolution velocity and saturation solubility. Stabilisers and polymers are usually required to prevent particle agglomeration and recrystallisation, ensuring physical stability during storage and administration. They are versatile NPs that can encompass semiconductor systems, displaying size-tunable fluorescence, photostability, and energy transfer efficiency, rendering them useful in fields other than nanomedicine [108].

Lopez-Vidal et al. [88] reported the development of pH-sensitive NCs coated with EL100-55, using ivermectin (IVM), a Biopharmaceutical Classification System (BCS) Class II compound, as the model drug. The design rationale aimed to enhance IVM’s solubility, with variable bioavailability and significant inter- and intra-individual variabilities. The choice of EL100-55 was based on its capacity to confer delayed-release properties, while protecting the drug from premature degradation. The effect of varying polymer concentrations (10%, 25%, 40%) on the NC’s PS, PDI, yield, and EE was investigated. All formulations presented PS lower than 500 nm, while the ones containing 25% and 40% of EL100-55 exhibited a more homogeneous PS distribution, with a PDI lower than 0.3. An increase in polymer content was also associated with higher EE; however, higher polymer content (40%) was associated with increased particle growth, suggesting a trade-off between EE and colloidal stability.

EE did not differ significantly among the formulations, with all NPs showing EE% values higher than 90%. These values remained practically unchanged over the 112 days stability period. This underscores efficiency of the technique in and producing coated particles, capable of achieving pH-dependent release. Additional stability studies were conducted for 3 months to evaluate whether the NCs underwent significant changes in PS and PDI during storage periods without protection from moisture or light. The sample with 25% polymer content exhibited a PS increase of approximately 30 nm, with no registered changes for PDI. On the other hand, the 40% formulation experienced an enlargement of around 75 nm. Additionally, the altered values still remained within an acceptable range. In vitro dissolution studies were conducted, simulating gastrointestinal pH shifts to study the influence of polymer coating, and comparing NCs containing 25% polymer with their physical mixture. In the first 2 h, both systems demonstrated less than 10% release (pH 1.2). As pH increased (pH 6.8), IVM’s release from the NCs rapidly occurred, whereas the physical mixture continued to show poor dissolution, with still less than 10% released even after pH adjustment. This disparity between systems confirmed that the polymer coating effectively shielded the drug from gastric acidity, enabling indirect selective delivery to the intestine due to EL100-55 ionisation. A second dissolution test (pH 7) was performed, adding sodium lauryl sulphate (SLS) to both formulations, in order to enhance IVM’s solubility and prevent medium saturation. In this case, the dissolution profiles of the coated NCs and the physical mixture did not differ so markedly. For the first 90 min, both formulations behaved similarly, gradually increasing drug release. For the following duration of the study, which was 180 min, the NCs were able to increase drug release when compared with the physical mixture. These results show that differences in surface tension between media with and without SLS further contributed to enhanced drug solubility, increasing released drug quantities. However, this also indicates that the performance advantage of the coating is most relevant under dissolution-limited conditions, whereas in solubilisation-enhanced media (with SLS), the formulation-dependent effects become less pronounced. For the coated NCs, drug release remained dependent on polymer disintegration prior to drug release, confirming the system’s pH-responsive behaviour. This enteric protection effectively delayed drug exposure until reaching intestinal conditions, resulting in a temporally controlled release profile. All in all, the NC’s solubility increased by 47-fold in water and 4.8-fold in simulated intestinal conditions (pH 6.8) compared to pure IVM [88].

Che et al. [89] developed nintedanib (BIBF) nanocrystals (BIBF-NCs) to lower the solubility of the drug in the stomach, thereby limiting premature gastric dissolution, while maintaining drug supersaturation in the intestine, thereby improving oral bioavailability. BIBF’s pH-dependent solubility, combined with drug recrystallisation in the intestinal lumen and P-gp-mediated efflux, contributes to its low oral bioavailability. NCs were prepared by using a hydrochloric acid solution at pH 1.2 as solvent, avoiding the use of organic solvents typically required for BIBF solubilisation. These NCs were coated with EL100, aiming to guarantee their stability. To obtain coated particles, freeze drying and spray drying were evaluated, using hydroxypropyl methylcellulose E5 (HPMC-E5) at different ratios as a protective agent to prevent aggregation. At HPMC-E5: drug ratios of 1:1 and 2:1, PS was preserved. However, excess HPMC-E5 slowed redispersion due to matrix formation around the crystals, physically blocking aggregation. Therefore, the chosen HPMC-E5: drug ratio to prepare coated BIBF-NC was 1:1. Dissolution assays demonstrated that uncoated BIBF-NC were completely dissolved at pH 1.2 within 2 h, whereas coated NCs with different polymer ratios avoided unwanted and early onset disintegration. Continuous increase in polymer concentration was responsible for the downward trend in NCs dissolution and drug release with a polymer: drug ratio of 5:1 enabling the reduction in drug release more significantly than all other ratios employed. No further ratios were tested because, although increasing polymer quantity reduces BIBF’s gastric dissolution, drug concentration would need to be decreased to maintain PS. The used drying technique also influenced NCs solubilisation, since at the same amount of Eudragit, spray-dried crystals presented a more significant acid-resistance, with lower drug release quantities. For this reason, the spray drying method, with a 5:1 ratio of EL100 to BIBF-NC, was selected as the final formulation process. PS stability of both coated and non-coated BIBF-NC was evaluated as well, under simulated physiological conditions, incubating them in pH 6.8 and 7.4 for 10 h. Uncoated NCs showed an increase in PS, which may be related to the pH and ionic strength sensitivity of SDS. For the coated crystals, HPMC-E5 played an important role in spatial stabilisation, maintaining the nanocrystalline PS. In vitro dissolution studies were performed for BIBF soft capsules, uncoated BIBF-NC, coated BIBF-NC (BIBF-NCs@L100), and their physical mixture, at pH 1.0 to pH 6.8, for 2 h. Due to BIBF’s pH-dependent solubility, results showed dissolution of BIBF in the stomach without the enteric material, with posterior precipitation in less acidic pH values. The presence of EL100 enabled a significant reduction in BIBF dissolution in the gastric environment. However, BIBF-NCs@L100 showed a cumulative dissolution of 36.43 ± 3.50%, while their physical mixture showed a value of 36.43 ± 4.97%. This suggests that the drug–polymer interactions were minimal and that the pH-dependent solubility is due to the spray-dried coating layer. An in vivo pharmacokinetic study was also performed on rats and included the utilisation of diluted powders with a BIBF concentration of 16.5 mg/mL. The formulations included uncoated BIBF-NC, its coated version, and BIBF soft capsules. The results showed that BIBF-NCs@L100 achieved the highest plasma concentrations, with Cmax and AUC values of 1.25- and 1.43-fold higher than those of uncoated NCs, respectively, and 2.59- and 2.58-fold higher than those of BIBF soft capsules, respectively. This enhancement was attributed to reduced PS, pH-dependent protection from gastric release, and maintenance of supersaturations, ultimately leading to superior oral bioavailability [89].

### 4.3. Lipid-Based Nanoparticles

Lipid-based NPs (LNPs) comprise a diverse group of lipid-based nanocarriers, including liposomes (LPs), solid lipid NPs (SLNPs), nanostructured lipid carriers (NLCs), and ionisable lipid NPs. These NPs have been extensively investigated and are widely utilised for the vehiculation of poorly water-soluble and highly lipophilic drugs, due to their ability to enhance drug solubility, stability, and bioavailability [109]. Due to their nanoscale size and unique physicochemical properties, these systems exhibit efficient cellular uptake and can improve the intracellular delivery of a broad range of therapeutic agents, including small molecules and biologically active compounds. Additionally, LNCs can be functionalized with targeting ligands, stimuli-responsive components, and polymer-based surface modifications, enhancing targeting precision, circulation time, and sophisticating therapeutic performance [110].

#### 4.3.1. Liposomes

LPs are spherical vesicles composed of lipids arranged in a unique bilayer structure, forming their outer layer, and surrounding an aqueous internal compartment. These NPs can carry both hydrophilic and hydrophobic drugs, while keeping a structural similarity with living cells, making them a promising DDS for a variety of therapeutic agents [111]. LPs are composed of single or multiple lipidic bilayers, derived from natural or synthetic phospholipids, and may contain unsaturated lipids, sphingolipids, steroids, glycosphingolipids, lipids conjugated to diene, methacrylate and thiol group or charge-inducing lipids [112]. LPs have already been studied for enteric drug delivery, mainly in the colonic environment. They have effectively interacted with both normal and inflamed colonic tissues in laboratory settings, strongly suggesting their potential as carriers for colon-targeted drug delivery [90].

A study conducted by Alghurabi et al. [90] sought to further assess Eudragit-coated bile salt-containing LPs (ES-SG/LP), through a combination of in vitro and in vivo experiments. 5-aminosalicylic acid (5-ASA), a compound characterised by low water solubility and limited permeability, primarily indicated to treat IBD, was selected as a model drug [113,114]. Bile salts (specifically sodium glycocholate) were incorporated into the liposomal bilayer for two main reasons: to promote membrane destabilisation upon contact with the intestinal epithelium, thereby facilitating vesicle uptake; and to stabilise the liposomal membrane against the disruptive effects of endogenous physiological bile salts. The inclusion of bile salts decreased PS and EE, with no effect on ZP. The reduction in EE was attributed either to competition between the anionic sodium glycocholate and 5-ASA for binding sites on the cationic lipid, or to destabilisation of the bilayer caused by the hydrophilic nature of sodium glycocholate, which may induce pore formation and facilitate drug leakage into the aqueous medium.

LPs with varying bile salt concentrations (0.25, 0.5, 1 mg/mL) were subsequently coated with ES100. The coating process resulted in increased NP’s PS values and negative ZP values, indicating reversal of the liposomal surface charge and confirming successful polymer deposition. Additionally, a slight increase in EE was also observed, which was attributed to the possible adsorption of 5-ASA beneath the polymeric layer.

Drug release behaviour was then evaluated for several liposomal formulations [LPs without bile salts, bile salt-containing LPs (SG/LP), and ES-SG/LP], under simulated gastrointestinal conditions, using three sequential biorelevant media, with pH values of 1.2, 5.0, and 7.4, Within the first 2 h, the LPs without bile salts had released approximately 60% of their 5-ASA content, whereas ES-SG/LP released only around 20% of 5-ASA, with the majority of drug release happening at pH 7.4. SG/LP showed slightly modified but significant release profiles compared to LPs without bile salts. After 5 h, ES-SG/LP showed a drug release of approximately 40%, confirming that the ES100 coating successfully inhibited release in an acidic environment, hence ensuring a more targeted delivery to the colon. As the therapeutic efficacy of 5-ASA is strongly dependent on the concentrations reached at the target site [115] the authors further examined cellular uptake as a determinant of local in vitro drug accumulation. LPs without bile salts, SG/LP, and ES-SG/LP were marked with fluorescein isothiocyanate, and all exhibited significantly improved cellular uptake than a fluorescein solution at pH 7.4, evidencing efficient cell membrane permeation and intracellular transport. Moreover, a pharmacokinetic assay, performed on male Wistar rats, demonstrated that ES-SG/LP reduced Cmax by about 37% compared with LPs without bile salts, while maintaining elevated plasma concentrations for up to 6 h. Furthermore, when comparing SG/LP and ES-SG/LP, the coated preparation reduced Cmax by approximately 15% comparatively to the uncoated one, underscoring the role of Eudragit in further delaying systemic absorption, favouring higher colonic fluorescein delivery [90].

The same authors developed a similar colon-targeted delivery system using liposomal carriers loaded with BUD and coated with ES100 (BUD-E-BSLPs). As before, the LPs were conjugated with bile salts to enhance stability and facilitate epithelial uptake. However, stearylamine was additionally included to impart a positive surface charge, enabling electrostatic attraction with anionic ES100, and improving coating efficiency. Importantly, stearylamine played a central role in modulating liposomal colloidal behaviour, as even low molar ratios markedly reduced aggregation, while intermediate concentrations maximised BUD EE, and excessive levels led to a decrease in encapsulation, suggesting membrane oversaturation and structural destabilisation. This was accompanied by a progressive increase in ZP from near-neutral values to highly positive surface charge, confirming charge inversion and saturation of cationic lipid incorporation.

To investigate coating conditions, the authors varied several parameters, including liposomal concentration (2, 4, 5, 7, 10 mM), ES100 concentration (0.25, 0.5, 1, 2, 2.5 mg/mL in 100 mM phosphate buffer, pH 8.0), and liposome/polymer volume ratio (1:1, 1:2, 1:3). The impact of these variables on PS, PDI, and ZP was evaluated. Two interactions between the LPs and ES100 occurred simultaneously: particle re-stabilisation, where charge reversal occurred, and full coating was achieved; or aggregation, which resulted from partially coated LPs interacting with uncoated ones, thereby increasing PS. A lower LP/ES100 ratio (1:1) was associated with a lower PS, owing to reduced lipid concentrations. Conversely, at a ratio of 1:3, an excess of ES100 was available, which led to an increased coating rate and minimal collisions between LPs. Similarly, enhanced polymer availability also promoted reduced PS by preventing aggregation and favouring re-stabilisation. Regarding the process, the rate of LP addition also affected coating success, since a slower addition allowed partially coated, negatively charged LPs to interact more efficiently with subsequently added cationic LPs. In contrast, faster addition rates led to simultaneous charge reversal across all particles, maintaining repulsive interactions between dispersed particles and reducing coating efficiency.

Beyond polymer effects, SG and cholesterol content introduced an additional layer of structural dependence that was strongly influenced by the manufacturing method. Increasing SG concentration generally promoted particle aggregation; however, this effect was significantly more pronounced in sonication prepared LPs compared with extruded systems, indicating that membrane organisation critically determines susceptibility to bile salt-induced destabilisation. While cholesterol content alone did not significantly affect PS, higher SG levels induced aggregation, whereas cholesterol rich compositions exhibited improved structural resistance. Notably, depending on the preparation method, SG exerted opposite effects on BUD EE: in sonicated LPs EE was reduced, whereas in extruded ones, EE was significantly increased. The presence of stearylamine dominated surface charge modulation, while SG influenced electrostatic properties, primarily in sonicated formulations, with negligible effects in extruded systems. Cholesterol molar ratio did not significantly alter ZP regardless of the preparation method, suggesting that electrostatic properties are primarily governed by cationic lipid incorporation rather than bilayer rigidity. Drug release studies were performed as well, for LPs without bile salts, SG/LP, BUD-E-LPs, and Bud-E-BSLPs, in media simulating different GIT regions: simulated gastric fluid pH 1.2 (SGF), fasted-state pH 6.5 (FaSSIF) and fed-state (FeSSIF) simulated intestinal fluids, and phosphate-buffered saline pH 7.4, representing the colonic conditions. In SGF, BUD-E-BSLPs inhibited approximately 85% of drug release within 2 h, compared with 65% of drug release from BS-LPs. In FaSSIF, around 60% of the drug was released from SG/LP after 4 h, whereas only 20% was released from BUD-E-BSLPs in the same timeframe. Both ES100-coated formulations, with and without bile salts (BUD-E-LPs and BUD-E-BSLPs), showed stability in FeSSIF, maintaining liposomal structure and size distribution. In this medium, BUD-E-BSLPs showed only 15% drug release after a 4 h incubation, while BUD-E-LPs released about 30%. Due to the lack of bile salts in its liposomal structure, BUD-E-LPs doubled drug release when compared to BUD-E-BSLPs. At pH 7.4, the two formulations showed similar release patterns, where about 65–85% was released within 24 h. Collectively, this data confirmed that drug release from BUD-E-BSLPs was pH-dependent, with minimal release under gastric and upper intestinal conditions, and marked increase at higher pH values, consistent with colon targeting. In a simulated colonic environment, the majority of the drug was released within 1 h.

Stability studies were performed as well, for 3 days and 5 weeks, at 25 and 4 °C, respectively, and further demonstrated that BUD-E-BSLPs exhibited a better capacity to prevent liposomal aggregation and destabilisation. The negative surface charge imparted by bile salts promoted electrostatic repulsion, which prevented liposomal aggregation during storage. Hence, overall, BUD-E-BSLPs showed better performance and stability across all in vitro studies [91].

#### 4.3.2. Solid Lipid Nanoparticles

SLNPs are nanoscale carriers built from a solid lipid matrix with a surfactant in the outer coat layer. The physical and chemical properties of these nanostructures are largely dictated by their constituent components, which in turn determine their biological behaviour. By adjusting their composition, these systems can fine-tune drug release patterns and influence the way a drug is distributed within the body, leading to improvements in solubility and overall bioavailability. SLNPs provide an attractive method for delivering drugs with precise control, longer bloodstream residency times, and reduced toxicity [116].

With the purpose of improving saxagliptin’s (SAX) oral bioavailability, Alhamhoom et al. [92] developed RS100-coated SLNPs loaded with SAX. This antidiabetic drug is characterised by limited membrane permeability, low aqueous solubility, and short elimination half-life (4–6 h), all of which contribute to its poor bioavailability. The use of SLNPs as a carrier allows bypassing hepatic first-pass metabolism and promotes lymphatic drug transport, thereby enhancing oral bioavailability. For successful encapsulation, SAX’s solubility in different lipids was first evaluated. Among the tested lipids, stearic acid exhibited the highest solubility and was therefore chosen for NP preparation. The optimised SLNPs were obtained using Poloxamer188 as an emulsifier and polyvinyl alcohol as a stabiliser, excipients that contributed to preventing particle aggregation and improving their stability. A Quality by Design approach, employing a Central Composite Design, was applied to optimise formulation and process variables, including lipid and polymer amounts, surfactant concentration, and homogenization speed. The obtained results demonstrated that lipid and polymer concentrations increased PS, likely due to increased viscosity and enhanced probability of particle aggregation, whereas higher surfactant concentrations and homogenization speeds reduced PS by improving emulsification efficiency and minimising agglomeration. EE was similarly dependent on formulation composition, increasing with higher lipid and polymer content due to greater drug accommodation within the lipid matrix. On the other hand, excessive surfactant concentrations negatively affected encapsulation, possibly by promoting drug partitioning into the aqueous phase.

In vitro diffusion studies were performed for pure drug and optimised SLNPs at pH 7.4. The SLNPs exhibited a slower, controlled release profile relative to the pure drug, which was attributed to the presence of RS100. The reduced RS100’s permeability limits the penetration of the dissolution medium into the NPs, thereby delaying drug dissolution and diffusion across the polymeric coating layer. Although the release data were fitted to the Korsmeyer–Peppas model, the authors did not use the release exponent, *n*, to classify the underlying drug-release mechanism. Subsequently, in vivo pharmacokinetic studies were performed in male albino Wistar rats, comparing pure SAX with the RS100 SLNPs. Results showed improved Cmax, increasing from 4.7 µg/mL to 5.1 µg/mL, delayed Tmax from 2 h to 3 h, and enhanced overall bioavailability, with AUC increasing from 78 to 112 ng·h/mL) for the coated SLNPs formulation, compared with pure SAX. In addition, the RS100 SLNPs exhibited lower elimination rate constants, confirming a more sustained drug release. Hence, RS100 enabled a controlled release profile due to low polymer permeability and, consequently, slow polymer swelling, which had a direct impact on the NPs’ biological performance [92].

Colonic drug delivery can also be attained using SLNPs. Using oxaliplatin (OXA) as the model drug, Golla et al. [93] developed ES100-coated SLNPs (OXA-ES100 SLNPs) for colorectal cancer treatment. Conventional OXA-based chemotherapy presents off-target toxicity on healthy cells and low bioavailability, therefore justifying the need for advanced delivery systems. Similar to what was done in the previous study, an experimental design methodology was applied to optimise OXA SLNPs. A Box–Behnken design was employed to investigate the influence of lipid, surfactant, and co-surfactant concentrations on PS and EE. These SLNPs were subsequently lyophilised and processed into pellets using an extrusion spheronization technique, after which they were spray-coated with ES100. The coating step contributed to an increase in PS and a decrease in EE, possibly due to partial OXA diffusion or surface-associated drug loss during this process. In vitro drug release was evaluated for two different pH values: gastric pH 1.2 and colonic pH 7.0. The release profiles demonstrated that OXA-ES100 SLNPs began releasing OXA after 4 h at neutral pH. At colonic pH, the percentage of OXA released from the coated NPs was significantly higher compared to the uncoated formulation. Since these two dissolution assays were performed separately, the reason for this difference lies in ES100’s insolubility at acidic pH, resulting in lower percentages of released OXA. These findings confirmed that OXA-ES100 SLNPs could successfully deliver OXA to the colon, indirectly achieving organ targeting through the pH-dependent solubility of ES100. Furthermore, the dissolution data suggest that drug release followed a controlled-release mechanism, where the polymer’s solubility properties regulated and sustained OXA’s release [93].

#### 4.3.3. Nanostructured Lipid Carriers

NLCs are advanced NPs built from a mixture of solid and liquid lipidic components. At physiological temperature, the lipids exist in either a solid or liquid state, creating a partially disordered internal structure that provides spaces within the lipid core, facilitating drug incorporation. The ratio and nature of the lipids forming NLCs will influence their configuration. Their structural irregularities reduce the risk of drug displacement, enabling higher drug loading compared with other nanoparticulate systems. These nanocarriers are transformed into chylomicrons following intestinal absorption, entering the lymphatic system, and promoting systemic delivery while bypassing first-pass metabolism, similarly to SLNPs [94]. Moreover, NLCs have demonstrated enhanced particle mucoadhesion, contributing to a prolonged residence time at the target site and, consequently, improved therapeutic efficacy [117,118].

Tacrolimus (TAC) is a calcineurin inhibitor used to manage refractory IBD. It is a significantly potent, water-insoluble drug that undergoes extensive first-pass metabolism. Aiming to achieve targeted colonic delivery, tacrolimus-loaded NLCs (TAC-NLCs) coated with FS100 (TAC-NLCs/E FS100) were designed. NLCs were obtained using the solid lipid compritol, the surfactant CTAB, and TAC as the model drug. FS100 was employed because it enables colon-targeted drug release and prevents premature degradation at gastric pH. When it reaches neutral pH, polymer swelling and dissolution occur, resulting in immediate drug release at the desired target site, optimisation of both TAC-NLCs and TAC-NLCs/E FS100 was also performed using the Box–Behnken design. Different NLC: FS100 ratios were studied to assess their impact on PS, ZP, and EE. The optimised formulation, containing a 1:2 ratio of NLCs to FS100, exhibited increased PS and ZP but reduced drug release at pH 1.2, likely due to limited penetration of the dissolution medium. A decrease in EE was also observed, potentially caused by drug migration from the lipid core to the outer aqueous phase, during the coating stage [94].

In vitro drug release of TAC-NLCs/E FS100 and TAC-NLCs, performed at pH 1.2, 4.5, and 7.4, using the dialysis bag diffusion method, revealed that TAC-NLCs/E FS100 exhibited controlled drug release over a 72 h period, with maximal release happening under colonic pH. Release kinetics analysis using the Korsmeyer–Peppas model indicated that uncoated NLCs followed Fickian diffusion, as suggested by an *n* value below 0.45. The initially observed burst release may be attributed to the rapid diffusion of drug molecules located near or at the surface of NLCs. Conversely, TAC-NLCs/E FS100 demonstrated non-Fickian release mechanism governed by polymer swelling and drug diffusion, consistent with the controlled release properties of RS100, and thus regulating burst release.

The in vitro cytotoxicity of TAC, TAC-NLCs, and TAC-NLCs/E FS100 (25, 50, and 100 µg/mL) was evaluated on thioglycolate elicited macrophages and colon cells, using the MTT assay. After 24 h of incubation, the viability of both cell types remained above 80% for all tested formulations, compared with the negative control Triton-X. Additionally, the results indicated that TAC-NLCs/E FS100 exhibited a favourable safety profile at all tested concentrations, with cell viability remaining independent of the applied concentration [94].

In vivo studies were performed as well, using a DSS-induced colitis rat model (the same model used by Raza et al.) [73]. Treatment groups received TAC, TAC-NLCs, or TAC-NLCs/E FS100. Among these, TAC-NLCs/E FS100 demonstrated superior therapeutic outcomes, with significantly greater body weight recovery and an improvement in survival rate of up to 80%. Biodistribution studies confirmed the presence and accumulation of the developed NLCs in the stomach, small intestine, and colon, with TAC-NLCs/E FS100 achieving markedly higher colonic drug concentrations. In contrast, and due to the initial burst release, TAC-NLCs exhibited increased drug content in the stomach [94].

Another colon targeting strategy was proposed by Borderwala et al. [95], using 5-fluorouracil (5-FUO) as the model drug. The clinical use of 5-FUO is limited by its short plasma half-life, and uncontrolled systemic concentrations, resulting in lack of therapeutic efficacy, and the need for frequent dose administration to maintain drug concentrations above the minimum effective level. These problems contribute to poor patient compliance, resistance development, systemic side effects, and drug distribution in high amounts to normal tissues. Intravenous administration often results in plasma concentrations above the maximum safe level, while oral administration is conditioned by first-pass hepatic and intestinal metabolisms, leading to unpredictable toxicity and efficacy. To overcome these limitations, 5-FUO-loaded NLC coated with ES100 (5-FUO-E-NLCs) were formulated. Regarding the optimised formulations, it was observed that the PS of 5-FUO-E-NLCs (154 nm) was higher than the PS of uncoated NLC (102 nm), and the ZP of uncoated NLC (−8.19 mv) was higher than that of coated NLC (−21.7 mv), attributable to the acrylic acids from the coating polymer. The coating process enhanced the EE of NLCs, with coated NLCs reaching 89.8%, compared to 83.5% for the uncoated NLCs. The 5-FUO release from a 5-FUO solution, 5-FUO NLCs, and 5-FUO-E-NLCs was evaluated in vitro, using pH variations to mimic the physiological GI transit. The 5-FUO solution burst-released the drug in the first 2 h at pH 1.2, while 5-FUO NLCs showed maximum release at pH 4.5, suggesting indirect intestinal targeting. The release of 5-FUO from coated NLCs followed a zero-order kinetics, and analysis using the Kornmeyer–Peppas model revealed a non-Fickian release mechanism. The presence of ES100 effectively controlled drug release, being in line with providing the colon with the largest drug concentrations, and achieving a spatial and temporal release pattern.

On the other hand, the performed ex vivo studies showed only partial and slow release of 5-FUO from 5-FUO solution, which was likely attributed to P-gp presence, which leads to drug efflux from the intestinal epithelium [115,119]. The release pattern of 5-FUO-E-NLCs in the performed ex vivo study was 7.40 ± 2.19%, 26.5 ± 4.45%, and 100.90 ± 3.96% after 2, 6, and 24 h, respectively. In vitro cytotoxicity of blank ES100-NLCs, 5-FUO NLCs, and 5-FUO-E-NLCs was carried out by MTT assay on Caco-2 cells. Blank ES100-NLCs led to a cell viability of 99%, indicating excipient safety. For the other formulations, cytotoxicity was dose-dependent, and cell viability was inversely proportional to 5-FUO concentration. 5-FUO NLCs and 5-FUO-E-NLCs exhibited higher cytotoxic activity, which could be associated with enhanced permeation and retention effect.

Pharmacokinetic studies, in albino Wistar rats, further demonstrated the advantages of the ES100 coating. 5-FUO was immediately released from the solution, represented by low Tmax, whereas both coated and uncoated formulations significantly prolonged 5-FUO’s Tmax, indicating their capacity to delay drug release. The relative bioavailability of 5-FUO NLCs and 5-FUO-E-NLCs were found to increase by 5 and 11-fold, respectively, compared to the 5-FUO solution [95].

### 4.4. Polymeric Nanoparticles

Polymeric NPs (PNPs) have become a cornerstone in modern drug delivery, primarily due to their customizable architecture and physicochemical adaptability. Their size ranges between 1 and 1000 nm, and they can be loaded with active compounds, entrapped within or surface-adsorbed to the polymeric core. Their advantages include controlled drug release, drug protection from the biological environment, and improved bioavailability and therapeutic efficacy. Frequently used polymeric materials include poly (lactic-co-glycolic acid) (PLGA), polyethylene glycol (PEG), poly (methyl methacrylate) (PMMA), and Eudragit, which offer tunable surface chemistry and mechanical properties, enabling precise modulation of drug release profiles [120,121].

In the context of ulcerative colitis therapy, Gao et al. [100] developed iridoid glycoside (IG)-loaded, dual-Eudragit-coated polymeric NPs (PLGA-E-PNPs), to improve IG’s oral bioavailability. The PNPs were designed using PLGA as a sustained release polymer, and ES100 and Eudragit L30-D 55 (EL30) as coating agents to overcome the limitations of single-triggered release systems. The aim of this dual-Eudragit formulation was to optimise colonic drug distribution by choosing the most advantageous EL30:ES100 ratio. These polymers were meant to prevent gastric drug release, minimising drug loss, and leading to higher colonic drug concentrations. The in vitro drug release of IG from PLGA-E-PNPs was evaluated in simulated physiological conditions along the GIT, along with two other nanoparticulate systems: IG-loaded pH-sensitive NPs (E-PNPs), containing EL30 and ES100, and IG-loaded PLGA NPs (TDNPs). In the first 6 h, less than 20% of IG had been released from E-PNPs and PLGA-E-PNPs, corresponding to inhibited drug release in the simulated stomach and intestinal fluids. TDNPs exhibited a burst drug release profile during the first 6 h, due to PLGA’s pH-independent properties, which corresponded to 70% of IG release. On the other hand, E-PNPs showed 100% burst release at pH 7.4, owing to the complete dissolution of ES100 and EL30D at pH > 7, whereas PLGA-E-PNPs maintained their intact NP morphology, suggesting no immediate colonic drug release. This was due to the presence of PLGA, which was able to sustain drug release after polymer dissolution when reaching pH 7.4.

After the in vivo therapeutic efficacy assessment of orally administered PLGA-E-NPs in a DSS-induced colitis rat model, the results showed a longer Tmax for PLGA-E-NPs than for free IG. Additionally, free IG achieved higher Cmax and AUC0-48 h values than the controlled targeted delivery NPs. The reason for this difference was the higher IG release and absorption, which occurred in the upper GIT. However, after 8 h, the drug’s poor stability and short half-life rendered it undetectable. On the contrary, PLGA-E-NPs exhibited a longer residence time, reaching their Cmax after 10 h, and maintaining high plasmatic concentrations during a 16 h period. By studying the different IG-containing formulations, the authors were able to assess that the dual polymeric strategy resulted in better performance outcomes than the other employed formulations. Burst release inhibition, increased colonic drug accumulation, and improved bioavailability of PLGA-E-NPs, with a 2.5-fold increase in AUC compared with the free drug, were key results that support the potential of dual-targeting PNPs [96].

Similar strategies were reported by Naeem et al. [122], who developed dual-targeting NPs destined for ulcerative colitis, using FS30D and PLGA as polymers to deliver cyclosporine A. For comparison, NPs containing only PLGA and only FS30 D were produced. All formulations exhibited equivalent PS (<300 nm), PDI (<0.1), and EE (>50%). Additionally, it was observed that particles maintained their size across different pH media mimicking the gastrointestinal tract. Regarding drug release at pH 1.2 and 6.8, formulations containing either FS30D alone or a mixture of PLGA and FS30D showed a similar profile, with less than 20% of drug release. In contrast, formulations with only PLGA released approximately 70% of the drug after 6 h. At pH 7.4 (simulating the ileum), differences emerged between the particles containing a mixture of polymers and NPs containing only FSD30D. Although this system employed only one Eudragit grade, the results were comparable: FS30D (as well as ES100 and EL30) controlled drug release time and location, while PLGA prevented burst release and provided sustained drug release after reaching pH 7.4, promoting higher drug concentrations in the desired targeted location, the inflamed colon. In vivo studies in mice with colitis demonstrated the superiority of NPs containing both polymers [122].

Together, these studies underscore the complementary role of Eudragit coatings in controlling release location and PLGA in prolonging drug release.

Beyond IBD, PNPs have also been employed to enhance the oral delivery of anticancer drugs. G. et al. [97] developed PNPs loaded with Dasatinib (DTN). DTN is a second-generation tyrosine kinase inhibitor, efficacious in targeting multiple tyrosine kinases associated with tumour cell proliferation. This drug’s oral bioavailability ranges from 14 to 34%, primarily due to its poor water solubility, short half-life of 3–5 h, and pH susceptibility, which complicate its effective delivery. Because of its weak basic nature, at a pH greater than 4, DTN’s solubility declines, meaning that DTN is more soluble in acidic pH [123]. This can lead to high gastric drug concentrations, which subsequently can result in gastric irritation and restricted drug absorption in the intestinal environment. DTN-loaded PNPs coated with EL100 (DTN-E-PNPs) were designed to achieve better tumour selectivity, minimise toxicity, control drug release, and increase DTN’s oral bioavailability. To optimise the synthesis of the PNPs, a 23 factorial design was employed. EL100 concentration, Poloxamer 407 concentration, and sonication power were the studied independent variables, whereas PS, ZP, and EE were the dependent ones. Among the several prepared batches, the optimised batch of DTN-E-PNPs included a drug–polymer ratio of 1:2, sonication power of 30 mV, and a Poloxamer 407 concentration of 1%. The in vitro drug release study was conducted for the optimised DTN-E-PNPs, and pure DTN, in two media (pH 1.2 and 6.8). Single DTN exhibited the lowest release profile, whereas DTN-E-PNPs significantly improved drug release. An initial burst release was registered for both formulations in the first hour. However, sustained drug release profiles were maintained throughout the following duration of the assay. Additionally, the release kinetics model that best fitted DTN-E-PNPs’ release profile suggested a diffusion-controlled mechanism, as indicated by the release exponent *n* = 405 in the Korsmeyer–Peppas model.

The cytotoxicity of single DTN and DTN-E-PNPs was evaluated in vitro, in MCF-7, MDA-MB-231, and 4T1 cell lines using the MTT assay. The used negative control was DOX, and the incubation period was 24 h. The results showed higher cytotoxic effects for DTN-E-PNPs when compared with DOX and DTN, with lower IC50 values, indicating a more potent anticancer activity. In vivo pharmacokinetics and biodistribution studies, in Wistar albino rats, confirmed the superiority of the nanoparticulate system, compared to the DTN. DTN-E-PNPs exhibited a Cmax of 12.60 ng/mL at 2 h (Tmax), an AUC0-t of 71.23 ng/mL·h, and an elimination half-life of 6.15 h, whereas for single DTN’s Cmax was 8.50 ng/mL at 4 h (Tmax), AUC0-t was 66.06 ng/mL·h, and the elimination half-life was 3.97 h. Furthermore, biodistribution assays revealed that both formulations accumulated in the spleen and kidney, though DTN-E-PNPs showed slightly reduced levels (2.83 ng/10 mg for the spleen, and 2.87 ng/10 mg for the kidney). The mammary tissue was not actively targeted, which indicates that the only mechanism targeting cancer cells was the passive EPR effect, leaving the mammary tissue with lower drug concentrations for both formulations (1.11 ng/10 mg for the DTN-E-PNPs). The formulation’s performance was studied in a rat mammary carcinoma model, which was 7,12-Dimethylbenz[a]anthracene (DMBA)-induced. The results supported the pharmacokinetic findings, since DTN-E-PNPs significantly reduced tumour volumes, improved survival rates, preserved body weight, and maintained healthier haematological parameters, compared to DMBA-treated and single DTN groups. The PNP’s remained stable in terms of PS, ZP, and EE for 6 months at 4.0 ± 1.0 °C, 25 ± 1.0 °C, and 40 ± 1.0 °C storage conditions. All in all, this work demonstrates that EL100-coated PNPs effectively suppressed gastric burst release, redirected drug release to the small intestine, and provided sustained delivery, thereby enhancing DTN’s oral bioavailability, stability, tolerability, and antitumour activity [97].

Beyond their classical coating function, Eudragit polymers have also been explored as structural components within nanoparticulate matrices. Studies report Eudragit-based NPs incorporating pH-independent polymers (RS100 and RL100), or hybrid polymer matrices.

For example, RS100 has been used to formulate nanoparticles for the delivery of eluxadoline, a drug used in the treatment of irritable bowel syndrome with diarrhoea. The release profile indicated a diffusion-controlled kinetic mechanism, as evidenced by its fit to the Higuchi equation [124]. In addition, RS100- and RL100-based nanoparticles loaded with phytochemicals have also shown release behaviour consistent with the Higuchi model [125]. In contrast, a recent study reported the development of E100- and silk fibroin-based NPs for the encapsulation of amphotericin, which exhibited a release profile consistent with the Korsmeyer–Peppas model [126].

These findings indicate that Eudragit can function not only as a surface modifier but also as a rate-controlling matrix, although this role remains less explored compared with its widespread use as an enteric coating.

#### Thiolated Nanoparticles

Thiolation represents a functional modification of Eudragit that extends its utility beyond conventional pH-dependent or release-controlling behaviour, by improving mucoadhesion, enhancing polymer–polymer interactions through disulphide bonding, and enabling redox-responsive release in physiologically reducing environments.

Thiolated NPs have been explored as redox-responsive drug delivery systems, enabling controlled release in reducing environments such as the colon, where bacterial metabolic activity lowers the redox potential, and in tumour cells, which are characterised by elevated intracellular glutathione levels [67,68,69,70,71].

NPs composed of thiolated RS100 and thiolated chitosan loaded with moxifloxacin were prepared with a mean PS of 87 nm, a PDI of less than 0.4 nm, and a spherical shape. Release of the drug from these NPs was compared with the reference product (Maxi tablets), and results demonstrated a more controlled drug release from the NPs. Moxifloxacin release from NPs was lower (range from 34% and 62%) in the absence of GSH than in the presence of GSH. Moxifloxacin release followed zero-order kinetics, while the release mechanism was consistent with Fickian diffusion, as indicated by fitting to the Higuchi equation and the Korsmeyer–Peppas model. In vivo studies in rats showed that these NPs enhanced the bioavailability of moxifloxacin more than 2-fold, as indicated by the increase in AUC. The NPs exhibited a Cmax of 876 ng/mL and a Tmax of 24 h, whereas the reference showed a Cmax of 678 ng/mL and a Tmax of 4 h [67].

Similarly, NPs composed of thiolated RS100, but with a different thiolated polymer (thiolated sodium alginate), were aimed at achieving enhanced colonic targeting by targeting tumour cells, which present higher levels of glutathione. NPs developed had PS between 103 nm and 145 nm, with a PDI ranging between 0.34 and 0.35. The smallest NPs had a rod shape and a rough texture. Release of paclitaxel was higher in the presence of GSH (69–95%) than in its absence (35–45%), confirming the activation by a reductive environment. Drug release from these NPs followed zero-order kinetics, with Fickian diffusion identified as the main release mechanism, as demonstrated by the Higuchi and Korsmeyer–Peppas models [68].

Additionally, aiming at the treatment of colorectal cancer, NPs of thiolated Eudragit RL100 and thiolated pectin were prepared, enabling dual encapsulation of aspirin and metformin. These NPs with a size smaller than 200 nm, presented a higher percentage of drug release in the presence of GSH. Consistent with studies involving thiolated Eudragit RS, these NPs released both drugs following zero-order kinetics, with the release mechanism fitting a Fickian diffusion model [69].

Another example of thiolated Eudragit-based NPs was prepared with a different thiolated Eudragit, namely thiolated ES100, combined with a thiolated poly(allylamine), enabling dual encapsulation of doxorubicin hydrochloride and curcumin with high EE, minimal release under physiological conditions but substantial release, above 90% for both drugs, under reductive conditions triggered by glutathione present on tumour cells [70]. Thiolated ES100 was also combined with thiolated dextran to prepare redox-sensitive NPs encapsulating doxorubicin. The study reported high EE and an approximately 80% drug release in a GSH-containing reducing media [71].

Collectively, these outcomes also illustrate that thiolation of Eudragit not only enhances encapsulation and stability compared with the unmodified polymer, but also imparts selective, redox-sensitive release profiles and superior oral bioavailability. Nevertheless, these benefits should be cautiously interpreted, as the reported performance depends on the extent of thiol functionalisation, the oxidation state of the thiol groups, and the surrounding biological environment, making their practical translation challenging. In addition, the added formulation complexity may limit reproducibility and scale-up, while robust in vivo validation remains limited.

## 5. Discussion

The development of oral DDS based on Eudragit-coated NPs represents a versatile strategy to address multiple limitations associated with conventional oral pharmaceuticals. The selection of a suitable Eudragit grade is a critical determinant in the rational design of oral nanoparticulate systems, as the polymer strongly influences the carrier’s physicochemical behaviour, as well as the site and kinetics of drug release. Across the diverse NPs considered in this review, formulation performance is governed by a non-linear interplay between surface chemistry, internal structure, and polymer-mediated release modulation, rather than by drug LC alone.

In MSNPs systems, aminomodification enhanced drug loading and coating efficiency by facilitating interaction with drugs containing hydrogen bond acceptor groups and promoting electrostatic interactions with anionic Eudragit polymers, namely ES100, as observed by Kassem et al. [85]. Adding to this, the authors observed that the increase in ZP caused by functionalisation further facilitated anionic polymer deposition. However, in this particular system, an adsorption saturation of the drug within the mesoporous network was noted, where higher NP concentrations did not correspond to an increase in adsorption efficiency. Morphology was also shown to critically influence formulation performance with dendritic architecture outperforming spherical and rod-shaped MSNPs in drug loading, pore accessibility, and mucoadhesion. Nonetheless, the same study also showed that exceeding the LC of MSNPs resulted in residual crystalline drug outside the pore structure, leading to burst release, which was a commonly observed phenomenon in this particular type of system. This shows that high drug loading does not necessarily translate into complete encapsulation of the drug within the mesopores. In parallel, they observed that Eudragit coating modified release kinetics through a shift towards non-Fickian diffusion, while Raza et al. [73] demonstrated that strong drug-silica interactions could substantially restrict release at intestinal pH, despite high EE. The polymer’s presence, in this particular case, also led to an increase in PS and aggregation, potentially compromising mucus penetration and scalability [75].

A similar non-linear formulation behaviour was also observed for NCs [88,89]. Lopez-Vidal et al. demonstrated that increasing stabiliser concentration did not proportionally improve nanosuspension properties, as the 1:2 drug/stabiliser ratio produced smaller particles and lower PDI values than the 1:3 formulation, suggesting that excess stabiliser may compromise milling efficiency or particle stabilisation [88]. Similarly, as observed by Jiajing Che et al., increasing polymer concentration during spray-drying coating did not directly translate into improved encapsulation or particle size reduction [89]. Despite improving gastric protection of the drugs, both these studies demonstrated that excessive coating may compromise redispersion, particle recovery, or dissolution behaviour.

Distinct formulation behaviours were observed in lipid-based systems due to the presence of a lipid matrix and the different roles assumed by Eudragit polymers. Considering SLNPs, Alhamhoom et al. demonstrated that increasing lipid and polymer concentrations increased PS and aggregation probability, likely due to reduced emulsification efficiency and increased viscosity during solvent injection preparation [89,92]. In contrast to liposomal systems, where higher polymer concentrations promoted stability and reduced PS through electrostatic stabilisation and membrane rigidification, in SLNPs the greater amount of lipid promoted rapid precipitation of lipid molecules, favouring larger particles. Nevertheless, higher lipid and polymer contents improved EE by increasing the available lipid matrix and reinforcing drug retention through the polymeric layer.

A central finding across all systems is that the functional role of Eudragit is not intrinsic but highly design-dependent, being dictated by the underlying NP architecture, drug physicochemical properties, and manufacturing process.

Regarding the manufacturing process, its selection depends not only on the physicochemical properties of the drug but also on the type of NPs intended to be obtained. Process parameters, such as time, temperature, and the use of organic solvents, play a decisive role in determining the critical quality attributes of the NPs and should therefore be carefully controlled to avoid compromising the specifications of the final drug product.

For instance, the use of a liquid CO_2_-assisted process was described as an appropriate alternative to formulate MSNPs loaded with MER and coated with ES100. This technique not only minimised the risk of residual organic solvents but also improved drug loading, ensuring a scalable and environmentally friendly strategy for oral NP production [73].

Moreover, the scalability of the manufacturing process is crucial for product development and industrial translation. Several methods have been described for the laboratory-scale production of Eudragit-based NPs; however, some studies have also addressed the potential scale-up of these processes.

Among the studies analysed, the microsphere-assisted nanomilling method employed for the nanocrystals production was highlighted as a scalable, top-down, organic solvent-free approach, capable of achieving favourable PS and PDI values [88]. Similarly, the production of Eudragit L100-coated nintedanib nanocrystals by nanomilling was reported as a process with promising scalability [89]. Nevertheless, the scalability of this process still needs to be confirmed through the production and evaluation of batches at an industrially relevant scale.

The scalability is also limited by the process safety concerns, particularly regarding the use of organic solvents. Therefore, for processes that rely on organic solvents, alternative solvent-free approaches can be explored to improve industrial viability, enhance environmental safety, and ensure product quality suitable for clinical application.

In line with safety considerations, when evaluating Eudragit-based NPs, it is important to assess the safety of the copolymers themselves. Fortunately, Eudragit polymers have a long record of safe use in conventional oral formulations, supported by more than 70 years of pharmaceutical application [127]. Their inclusion in pharmacopeial monographs further underscores their regulatory acceptance. Additionally, as outlined in Table 1, the FDA Inactive Ingredient Database provides values for the Maximum Daily Exposure (MDE) and/or Maximum Potency per Unit Dose (MPD) for oral administration of conventional dosage forms containing Eudragit polymers [79,80].

For instance, EL30-55 has been incorporated into the FDA-approved Procysbi^®^ for nephropatic cystinosis, where it functions as a protective coating. Toxicological evaluations of EL30-55, including 6-month and 1-year oral studies in rats and dogs, have demonstrated no significant adverse effects at doses up to 600 mg/kg/day and 80 mg/kg/day, respectively [128]. However, the application of these polymers in nanoparticulate systems necessitates additional safety evaluations due to the unique physicochemical properties of NPs. In vitro evaluations have shown that Eudragit-coated NPs generally maintain low toxicity at relevant concentrations, with surface charge and surfactant composition modulating cellular responses [129]. An in vitro comparative study assessing RL100, chitosan, PLGA, and poly-ε-caprolactone-based NPs revealed that RL100-based formulations displayed the highest cytotoxicity and haemolysis, effects attributed to the presence of quaternary amine groups, which can induce cytotoxicity, suppress DNA replication, and trigger inflammatory responses [130,131]. Another research study showed the implications of chronic exposure to different polymeric NPs on the biological function of Drosophila melanogaster. The studied polymeric NPS were coated with different coatings: polysorbate 80, PEG, chitosan, and RS100. When employing 500 µL doses, all polymers showed potential toxicity. However, the RS100-based NPs caused the most changes, even at doses of 100 µL. Notably, cationic NPs have more extensive toxicity than anionic ones, due to electrostatic interactions with negatively charged cell membranes [132,133]. Hence, these findings emphasise the importance of systematic toxicological assessments, particularly given the need for chronic dosing in many of the therapeutic indications targeted by oral NPs.

The patent landscape reflects the growing pharmaceutical interest in Eudragit-coated NPs for oral drug delivery and highlights key priorities for clinical translation. Patents such as US 9,700,544 B2 [134] and US 10,391,059 B2 [135] from Rapamycin Holdings, disclose rapamycin NPs protected by ES100 for gastric stability and intestinal release. US 9,452,930 B2 [136], which describes a continuous polymer-coating process with supporting data on RL100-coated NPs, underscores the importance of scalable and reproducible manufacturing methods. Additional filings highlight therapeutic diversification. For example, US 20130034602 A1 [137] discloses enteric-coated capsules containing insulin-loaded cationic NPs protected with Eudragit-based coatings. Similarly, CN 108379560 A [138] describes insulin NPs coated with Eudragit for enteric solubility control. More recent applications, such as US 20180318230 A1 [139], focus on blended systems, including PLGA-PEG/Eudragit NPs with pH-triggered oral insulin release. Collectively, these patents demonstrate the versatility of Eudragit across therapeutic areas, while also illustrating the dual emphasis on functional performance and scalable production technologies.

The translation of these nanosystems into clinical applications remains a significant gap. Although numerous preclinical studies have investigated Eudragit-based or Eudragit-coated NPs for oral delivery, their clinical translation remains limited. One human pharmacokinetic study evaluated etoricoxib-loaded PLGA NPs incorporated into Eudragit S100-coated capsules in six healthy volunteers. The NP-based formulation was compared with the marketed reference product and showed improved oral bioavailability of etoricoxib. Nevertheless, broader clinical evaluation is still required to establish the long-term safety, reproducibility, and therapeutic relevance of Eudragit-associated nanoparticulate systems [140].

Finally, to advance the development of a new oral drug product based on Eudragit^®^-based NPs, the formulation must be developed in accordance with relevant regulatory expectations, particularly those established by the EMA and FDA. In the context of nanotechnology, EMA “Nanotechnology-based medicinal products for human use” provides important considerations for the development, characterisation, quality control, safety assessment, and clinical evaluation of nanomedicines [141]. This addresses different types of nanotechnology-based systems, including inorganic NPs, nanocrystals, lipid-based NPs, and polymeric NPs, and is therefore relevant for the further optimisation of Eudragit^®^-based nanocarriers.

## 6. Conclusions

Altogether, the evidence indicates that Eudragit-coated NPs hold significant promise in the rational design of next-generation oral therapies. The small intestine and colon remain the primary target sites, which explains the frequent use of Eudragit L and S grades in formulations for IBD and colorectal cancer. Beyond site-specific targeting, these polymers are also employed to improve the solubility of poorly water-soluble drugs, thereby broadening their therapeutic applicability. Among the various delivery platforms, polymeric NPs have emerged as the most widely used carriers, underscoring their versatility and compatibility with Eudragit-based coatings.

Further knowledge is still needed regarding the mechanisms involved in the development of Eudragit-based nanoparticulate systems. In particular, additional studies are required to better understand the critical material attributes that influence formulation optimisation, thereby generating systematic information to support manufacturing scale-up and clinical translation.

## Figures and Tables

**Table 2:** Outline of selected Eudragit-containing nanoparticulate systems.

Nanoparticle Type	Drug	Eudragit Grade	Production Method	Physicochemical Properties		Target Site	Reference
				Uncoated	Coated		
Mesoporous Silica Nanoparticles	Catechin	Eudragit S100	Rotary evaporation method followed by polymeric coating	PS: 63.86 nm ZP: −34.25 mV PDI: 0.08 ± 0.03 LC: 15.24% EE: 53.95%	PS: 159.67 nm ZP: −40.25 mV PDI: 0.23 ± 0.09 LC: 21% EE: 82%	Colon	[85]
	Budesonide	Eudragit S100	CTAB-templated method followed by polymeric coating	Spherical PS: 110 nm (); ZP: −28 ± 0.38 mV EE: 44% Rod-like PS: 115 × 45 nm (); ZP: 21.61 ± 1.35 mV EE: 44% Dendritic PS: 100 nm −22.23 ± 1.21 mV EE: 44%	NA	Colon	[86]
	Prednisolone and Budesonide	Eudragit S100	Rotary evaporation method followed by polymeric coating	Pred PS: 160.1 ± 14.6 nm ZP: NA PDI: 0.21 ± 0.04 BUD PS: ≈238 nm ZP: −19.2 ± 2.8 mV PDI: 0.42 ± 0.11 LC: 95.2%	Pred PS: ≈238 nm ZP: −19.2 ± 2.8 mV PDI: 0.42 ± 0.11 EE: 95.2% BUD PS: ≈242 nm ZP: −19.5 ± 3.5 mV PDI: 0.45 ± 0.16 EE: 82.0%	Colon	[87]
	Meropenem	Eudragit S100	CTAB-templated method followed by polymeric coating	PS: 141 nm ZP: NA	PS: 645 nm ZP: −9 mV LC: 24.1%	Small intestine	[73]
Nanodiamond-Based Nanoparticles	Doxorubicin	Eudragit S100	Polydopamine coating followed by solvent evaporation	PS: 183.43 ± 6.62 nm ZP: 15.4 ± 0.3 mv PDI: 0.249	PS: 221.4 ± 5.86 nm ZP: −28.1 ± 0.7 PDI: 0.207 LC: 12.86 ± 0.61%	Colon	[64]
Nanocrystals	Ivermectin	Eudragit L100-55	Wet milling, followed by spray drying	PS: 198 ± 1 nm PDI: 0.184	PS: 294 ± 4 nm PDI: 0.278 EE: 92.3 ± 1.23% **	Small intestine	[88]
	Nintedanib	Eudragit L100	Wet milling followed by spray drying	PS: 290.80 ± 5.05 nm ZP: −41.93 ± 0.60 mv PDI: 0.103 ± 0.034	PS: 295.72 ± 3.04 nm ZP: −34.33 ± 0.68 mV PDI: 0.186 ± 0.054	Small intestine	[89]
Liposomes	5-aminosalicylic acid	Eudragit S100	Thin-film hydration followed by probe sonication and layer-by-layer electrostatic deposition	PS: ≈100 nm ZP: ≈60 mv PDI: ≈0.1	PS: ≈240 nm ZP: ≈ −26 mV PDI: ≈0.25 EE: ≈20% *	Colon	[90]
	Budesonide	Eudragit S100	Thin-film hydration followed by probe sonication and layer-by-layer electrostatic deposition	NA	PS: 275 nm ZP: −38 mV PDI: 0.128	Colon	[91]
Solid Lipid Nanoparticles	Saxagliptin	Eudragit RS100	Modified solvent injection	NA	PS: 212 to 442 nm ZP: −41.09 ± 0.11 to 30.86 ± 0.63 mV PDI: 0.26 ± 0.051 to 0.45 ± 0.017	Small intestine	[92]
	Oxaliplatin	Eudragit S100	Solvent emulsification followed by pelletisation	PS: 96.02 ± 1.45 nm EE: 89.32 ± 0.14%	PS: 116.81 ± 1.37 nm EE: 81.12 ± 0.26%	Colon	[93]
Nanostructured Lipid Carriers	Tacrolimus	Eudragit FS100	Modified microemulsion method followed by polymeric coating	PS: 170.4 nm ZP: 42.4 mv EE: 90%	Size: 198.7 nm ZP: −47.6 mV PDI: 0.176 EE: 78%	Colon	[94]
	5-Fluorouracil	Eudragit S100	High-pressure homogenisation followed by polymeric coating	PS: 101.7 ± 1.32 nm ZP: −8.19 ± 1.03 mV PDI: 0.27 ± 0.02 EE: 83.50 ± 2.4%	PS: 154 ± 3.17 nm ZP: −21.7 ± 2.02 mV PDI: 0.29 ± 0.07 EE: 89.81 ± 2.6%	Colon	[95]
Polymeric Nanoparticles	Iridoid glycoside	Eudragit S100 Eudragit L30-D 55	Single emulsion solvent evaporation followed by polymeric coating	NA	PS: 247 ± 26 nm ZP: −22.4 ± 1.76 mV PDI: 0.21 ± 0.05 EE: 39.47 ± 2.69%	Colon	[96]
	Dasatinib	Eudragit L100	Single emulsion solvent evaporation followed by polymeric coating	NA	PS: 202.1 ± 5.7 nm ZP: −18 ± 1.01 mV EE: 93.11 ± 0.2%	Small intestine	[97]

* values of ES100-coated LPs with 1 mg/mL of sodium glycocholate; ** Polymer concentration of 25%. BUD: Budenoside; CTAB: cetyl trimethyl ammonium bromide; EE: encapsulation efficiency; LC: loading capacity; NA: not available; PDI: polydispersity index; Pred: prednisolone; PS: Particle size; ZP: Zeta potential.

## Data Availability

No new data were created or analysed in this study. Data sharing does not apply to this article.

## Abbreviations

The following abbreviations are used in this manuscript:

5-ASA	5-Aminosalicylic acid
5-FUO	5-Fluorouracil
5-FUO-E-NLCs	5-Fluorouracil-loaded, Eudragit-coated nanostructured lipid carriers
API	Active pharmaceutical ingredient
AUC	Area under the curve
AZM	Acetazolamide
AZM-SH	Acetazolamide derivative
BCS	Biopharmaceutical Classification System
SG/LP	Bile salt-containing liposomes
BIBF	Nintedanib
BIBF-NCs	Nintedanib nanocrystals
BIBF-NCs@L100	Eudragit-coated BIBF-NCs
BUD	Budenoside
BUD-A-MSNPs	Budesonide-loaded, amino-functionalised MSNP
BUD-A-E-MSNPs	Budesonide-loaded, Eudragit-coated, amino-functionalised MSNP
BUD-E-LPs	Budesonide-loaded, Eudragit-coated liposomes
BUD-E-BSLPs	Budesonide-loaded, bile salt-containing liposomes
CHT	Catechin
CTAB	Cetyl trimethyl ammonium bromide
DAI	Disease Activity Index
DMBA	7,12-Dimethylbenz[a]anthracene
DOX	Doxorubicin hydrochloride
DDS	Drug delivery system
DSS	Dextran sulphate sodium
DTN	Dasatinib
DTN-E-PNPs	Dasatinib-loaded, Eudragit-coated polymeric NPs
ES@PND-PEG-TPP/DOX	Eudragit-coated PND-PEG-TPP/DOX
EE	Encapsulation efficiency
EL100	Eudragit L100
EL100-55	Eudragit L100-55
EL30D	Eudragit L30D-55
EGFR	Endothelial growth factor receptor
E-PNPs	Iridoid glycoside-loaded pH-sensitive polymeric NPs
EPR	Enhanced permeability and retention
ES100	Eudragit S100
ES-SG/LP	Eudragit-coated, bile salt-containing liposomes
E@MSND-BUD	Enteric-Coated MSNDs-BUD
Eud-MER-MCM-NH_2_	Eudragit-coated, MER-MCM-NH_2_
FaSSIF	Fasted-state simulated intestinal fluid
FDA	Food and Drug Administration
FeSSIF	Fed-state simulated intestinal fluid
FS30D	Eudragit FS30D
FS100	Eudragit FS100
GIT	Gastrointestinal tract
hCA IX	Human Carbonic Anhydrase IX
HPMC-E5	Hydroxypropyl methylcellulose E5
IBD	Inflammatory bowel disease
IG	Iridoid glycoside
INP	Inorganic NP
IVM	Ivermectin
LNP	Lipid-based NP
LC	Loading capacity
LP	Liposome
MCM	Mesoporous silica NPs MCM 41
MCM-NH_2_	Amino-functionalised MCM
MER	Meropenem
MER-MCM	MER-loaded-MCM
MER-MCM-NH_2_	MER-loaded-amino-functionalised MCM
MER-MCM-PO_3_	MER-loaded-phosphonate functionalised MCM
MTT	3-(4,5-dimethylthiazol-2-yl)-2,5-diphenyltetrazolium bromide
MSNP	Mesoporous silica NP
MSND	Dendritic MSNP
MSND-BUD	BUD-loaded MSND
NC	Nanocrystal
ND	Nanodiamond
NDNP	Nanodiamond-based NP
NH_2_-MNSPs	unloaded amino-functionalized MSNPs
NH_2_-MNSPs@EUS-100	NH2-MNSPs coated with ES100
NH_2_-MSNPs/CHT	Catechin-loaded NH2-MNSPs
NH_2_-MSNPs/CHT@EUS-100	NH2-MNSPs/CHT coated with ES100
NIR	Near-Infrared
NLC	Nanostructured lipid carrier
NP	Nanoparticle
MSNPs/CHT	Catechin-loaded MNSPs
OFLO	Ofloxacin
OXA	Oxaliplatin
P-gp	P-glycoprotein
PCR	Polymerase chain reaction
PDI	Polydispersity index
PEG	Polyethylene glycol
PLGA	Poly (lactic-co-glycolic acid)
PLGA-E-PNPs	Iridoid glycoside-loaded, Eudragit-coated, PLGA-based polymeric NPs
PMMA	Poly (methyl methacrylate)
PM	Physical mixture
PND	ND coated with polydopamine
PND-PEG	PEG-Functionalized PND
PND-PEG-TPP	TPP-functionalized PND-PEG
PND-PEG-TPP/DOX	Doxorrubicin-loaded PND-PEG-TPP
PNP	Polymeric NP
Pred	Prednisolone
Pred-A-MSNPs	Prednisolone-loaded, amino-functionalised mesoporous silica NPs
Pred-A-E-MSNPs	Prednisolone-loaded, Eudragit-coated, amino-functionalised mesoporous silica NPs
PS	Particle size
RS100	Eudragit RS100
SAX	Saxagliptin
SEM	Scanning electron microscopy
SLNP	Solid lipid NPs
SLS	Sodium lauryl sulphate
SGF	Simulated gastric fluid
TAC	Tacrolimus
TAC-NLCs	Tacrolimus-loaded nanostructured lipid carriers
TAC-NLCs/E FS100	Tacrolimus-loaded, Eudragit-coated nanostructured lipid carriers
TDNPs	Iridoid glycoside-loaded time-dependent polymeric NPs
TEM	Transmission electron microscopy
TEOS	Tetraethoxysilane
TPP	Triphenylphosphonium
ZP	Zeta potential
